# Single cell genomics as a transformative approach for aquaculture research and innovation

**DOI:** 10.1111/raq.12806

**Published:** 2023-03-07

**Authors:** Rose Ruiz Daniels, Richard S. Taylor, Diego Robledo, Daniel J. Macqueen

**Affiliations:** ^1^ The Roslin Institute and Royal (Dick) School of Veterinary Studies The University of Edinburgh Midlothian UK

**Keywords:** aquaculture, bioinformatic pipelines, cell isolation, nuclei isolation, single cell genomics, transformative applications

## Abstract

Single cell genomics encompasses a suite of rapidly maturing technologies that measure the molecular profiles of individual cells within target samples. These approaches provide a large up‐step in biological information compared to long‐established ‘bulk’ methods that profile the average molecular profiles of all cells in a sample, and have led to transformative advances in understanding of cellular biology, particularly in humans and model organisms. The application of single cell genomics is fast expanding to non‐model taxa, including aquaculture species, where numerous research applications are underway with many more envisaged. In this review, we highlight the potential transformative applications of single cell genomics in aquaculture research, considering barriers and potential solutions to the broad uptake of these technologies. Focusing on single cell transcriptomics, we outline considerations for experimental design, including the essential requirement to obtain high quality cells/nuclei for sequencing in ectothermic aquatic species. We further outline data analysis and bioinformatics considerations, tailored to studies with the under‐characterized genomes of aquaculture species, where our knowledge of cellular heterogeneity and cell marker genes is immature. Overall, this review offers a useful source of knowledge for researchers aiming to apply single cell genomics to address biological challenges faced by the global aquaculture sector though an improved understanding of cell biology.

## 1 INTRODUCTION

The field of genomics is rapidly transitioning from a state where bulk samples are predominantly studied, providing read‐outs averaged across all cells, to a position where the molecular profiles of individual cells can be reliably profiled. As most phenotypes result from the actions and interactions of specific cell types, generating cell‐specific rather than tissue‐averaged molecular data creates a large up‐step in information on the mechanisms underpinning trait expression. Such knowledge will have great value in research applications addressing biological challenges faced by the aquaculture sector, representing ongoing barriers to the sustainable production of many species, including, for example, the threat posed by disease outbreaks caused by diverse parasites and pathogens.

Single cell genomics involves the molecular analysis of individual cells, typically using high‐throughput sequencing technologies.^1^ The transformative impact of such methods on our understanding of cellular and developmental biology, immunology and disease biology in humans and model organisms cannot be overstated.^2^, ^3^, ^4^ The most common application is single cell transcriptomics, though methods for profiling epigenomic features at single cell resolution, inclusive of methylation (e.g., Ref. 5), chromatin accessibility (e.g., Ref. 6) and chromosome conformation,^7^ are maturing rapidly,^8^ as is single cell proteomics.^9^ In model organisms, it is now common to combine different single cell technologies to generate ‘multi‐omic’ profiles from the same cells.^10^ The scale of recent knowledge advancement in this field is perhaps best illustrated by the Human Cell Atlas initiative, which aims to deliver comprehensive molecular maps of all human cell types.^11^, ^12^, ^13^, ^14^

This review concerns the uptake and applications of single cell genomics in aquaculture research, a relatively new field that is expanding rapidly. Our primary focus is on single cell transcriptomics (Section 2), performed using single cell (sc) or single nuclei (sn) RNA‐Sequencing (scRNA‐seq and snRNA‐seq, respectively) on various platforms (reviewed in Ref. 15; also see Section 2) that have increased dramatically in throughput over recent years, such that thousands to hundreds of thousands of cells are being routinely profiled in single studies.^16^ Compared to bulk transcriptomics using RNA‐seq, which is already widely utilized in aquaculture research (reviewed in Ref. 17), sc/snRNA‐seq is a ‘game changer’ due to its ability to identify cell types and their heterogeneity, along with resolving cell‐specific gene expression responses to environmental perturbation.^18^ Such data are especially informative in species where knowledge of cellular biology is limited, that is, most aquaculture species.

While confined to a few labs in its early applications, extensive development of methods and commercial platforms has brought single cell technologies within the reach of many researchers.^16^, ^18^ A portfolio of single cell transcriptomic studies have recently emerged in non‐model aquatic taxa, including Atlantic cod,^19^, ^20^ Mexican cavefish,^21^ corals,^22^ and diverse aquaculture species, including Atlantic salmon,^23^, ^24^ rainbow trout,^25^, ^26^ orange‐spotted grouper,^27^, ^28^ Nile tilapia,^29^, ^30^ kuruma shrimp^31^ and Pacific oyster,^32^ among others (Table 1). This growing body of work immediately demonstrates the potential of single cell transcriptomics to transform our understanding of cellular biology in aquaculture species (Section 3), in settings that can be exploited to address diverse sustainability challenges,^33^ including the pressing need to manage disease outbreaks.^34^

**TABLE 1:** Summary of single cell transcriptomic studies in aquaculture species including major knowledge advances

Species	Sample type	Approach	Summary and knowledge advances	Citations
Atlantic salmon	Liver	snRNA‐seq 10x platform	Liver cell atlas capturing host response to bacterial infection; revealed unique hepatocyte states associated with infection and resolved major immune cell types	24
	Gill	snRNA‐seq 10x platform	Gill cell atlas; revealed known and novel gill cell types and cellular remodelling during smoltification, including evidence for repression of immune system	23
Rainbow trout	Blood	scRNA‐seq 10x platform	Atlas of sorted B cell heterogeneity; improved understanding of B cell heterogeneity, including new marker genes for distinct B cell subsets and states	26
	Blood	scRNA‐seq 10x platform	Analysis of antibody gene diversity in sorted B cells; supporting hypothesis that trout B cells produce antibodies with distinct specificities	25
Turbot	Multi‐tissue	scRNA‐seq 10x platform	Immune cell atlas; identified sixteen immune cell sub‐types, including major classes of lymphocytes and myeloid cells	64
	Multi‐tissue	scRNA‐seq 10x platform	Immune cell atlas; identified fourteen immune cell sub‐types, including major classes of lymphocytes and myeloid cells; providing evidence that neutrophils are key effectors of trained innate immunity	66
Orange spotted grouper	Midbrain	scRNA‐seq 10x platform	Midbrain cell atlas capturing response to RGNNV infection; described thirty‐five cell types, both neuronal and non‐neuronal, with evidence that microglia transform into macrophages upon infection	27
	Gonads	scRNA‐seq 10x platform	Cell atlas of adult male gonad; identified 10 cell types, including germ and somatic cells, with a panel of associated cell‐specific marker genes	28
Nile tilapia	Head kidney	scRNA‐seq 10x platform	Atlas of immune cells; revealing major populations of lymphocytes and myeloid cells, including diverse B and T cell subsets	29
	Head kidney	scRNA‐seq 10x platform	Atlas of immune cells after viral stimulation; revealed major populations of lymphocytes and myeloid cells, and subsets of non‐specific cytotoxic cells (akin to natural killer cells)	30
Asian sea bass	Ovaries	scRNA‐seq Drop‐seq	Adult cell atlas; identified multiple germ cell and somatic cell types and revealed conserved and divergent ovary development mechanisms via comparative single cell transcriptomics	44
Kuruma shrimp	Haemolymph	scRNA‐seq Drop‐seq	Atlas of cell types; described six haemocyte subtypes and their marker genes, and evidence for factors driving haemocyte differentiation	31
White shrimp	Haemolymph	scRNA‐seq 10x platform	Atlas of cell types; described haemocytes, monocytic haemocytes and granulocytes, including macrophage‐like haemocytes	65
Black tiger shrimp	Hepatopancreas Haemolymph	scRNA‐seq 10x platform	Atlases of cell types; identified seven haemocyte sub‐types including candidate stem cells and seven cell types in hepatopancreas, along with cell‐resolved responses to ammonia nitrogen stress	188
Hong Kong Oyster	Haemolymph	scRNA‐seq 10x platform	Atlas of haemocytes; identified thirteen populations including granulocytes, semi‐granulocytes, and hyalinocytes, revealing candidate transcription factors driving granulocyte lineage differentiation.	189
	Haemolymph	scRNA‐seq 10x platform	Investigated cellular heterogeneity in response to copper exposure, revealing cell‐specific sensitivities to and markers for copper exposure	32

However, single cell genomics is more challenging than equivalent bulk methods in multiple respects, presenting a barrier to broad‐scale uptake in aquaculture research. This includes the lab work, requiring high‐quality cells or nuclei and specialist library preparation methods, which brings several considerations to achieve optimal results (e.g., Refs. 35, 36) (see Section 4). Optimization efforts in single cell genomics have concentrated on mammals, which have major differences in biology with aquatic species. Analysis of single cell data is more complicated than bulk genomics due to its higher dimensionality. Multiple decisions are involved at many steps of data analysis, with a plethora of tools available, but without tried‐and‐tested standards that fit all studies, species and problems.^37^, ^38^ This challenge is compounded in aquaculture species as analysis pipelines are optimized for model species (see Section 5). Furthermore, while reference genomes are now available for many aquaculture species,^39^ they remain comparatively poorly annotated, which is one of several challenges faced when the aim is to transfer knowledge on cell biology across species—a standard practise to classify cell types on the basis of known marker genes from well characterized organisms.

This review starts with a brief overview of single cell transcriptomics (Section 2), before outlining the potential applications and impacts that single cell genomics can bring to aquaculture research (Section 3). Subsequently, we explore barriers to achieving such impacts, along with potential solutions and considerations when designing/executing experiments relevant to the wet‐lab work (Section 4) and also concerning the downstream data analysis and interpretation (Section 5). The overall aim is to provide a state‐of‐the art review on single cell genomics relevant to aquaculture researchers, while also offering recommendations and tips for those aiming to uptake single cell methods in their research.

## 2 WHAT IS SINGLE CELL/NUCLEI TRANSCRIPTOMICS?

In a nutshell, single cell transcriptomics involves the global profiling of gene expression in individual cells or nuclei. It is not our aim to describe the development of this field (see reviews by Refs. 1, 4), nor do we comprehensively review the various platforms currently on offer (see Ref. 40). Instead, this section offers a short primer on single cell transcriptomics to provide context for the remainder of the review.

Currently the most popular high‐throughput single cell transcriptomics methods are droplet‐based, with all studies published to date in aquaculture species using such strategies (Table 1). These approaches employ microfluidic capture of cells/nuclei inside microdroplets that contain an mRNA capture bead that includes a unique barcode, typically next to a unique molecular identifier (UMI).^41^ Subsequently, reverse transcription is used to generate a cDNA product with the expressed transcript linked to the barcode and UMI. In downstream analysis, the cell barcode retains the identity of the captured cells or nuclei, while the UMI ensures only unique transcripts are quantified. The resulting cDNA is used to make a library for sequencing, typically on a high‐throughput short‐read platform, which may be indexed to distinguish different samples. An important consideration at this stage is sequencing depth per cell/nuclei, a product of the (per sample) output of sequencing divided by the number of cells or nuclei captured, with 25,000–100,000 reads per cell/nucleus offering sufficient depth for most applications.

The most widely used single cell transcriptomics platform is the 10x Genomics Chromium, which is user‐friendly with demonstrated capability to detect a high number of transcripts per cell in diverse taxa and sample types. The popularity of this platform extends to most studies performed with aquaculture species to date (e.g., Table 1). However, it is also the most expensive droplet based approach on the market, with other available platforms being more affordable, including DropSeq^42^ (commercialized by Dolomite Bio) and InDrop.^43^ Studies have validated DropSeq in several commercially important aquatic species.^19^, ^31^, ^44^ There also exist non‐droplet based approaches including Smart‐seq2,^45^ where single cells are separated with a micro‐capillary pipette or via FACS and then individually sequenced, along with the microplate‐based method SPLiT‐seq,^46^ where cells or nuclei are uniquely labelled through multiple barcoding rounds. This strategy has been commercialised by Parse Biosciences, with the advantage that no platform or FACS sorting is required.

## 3 APPLICATIONS OF SINGLE CELL GENOMICS IN AQUACULTURE RESEARCH

**FIGURE 1:**
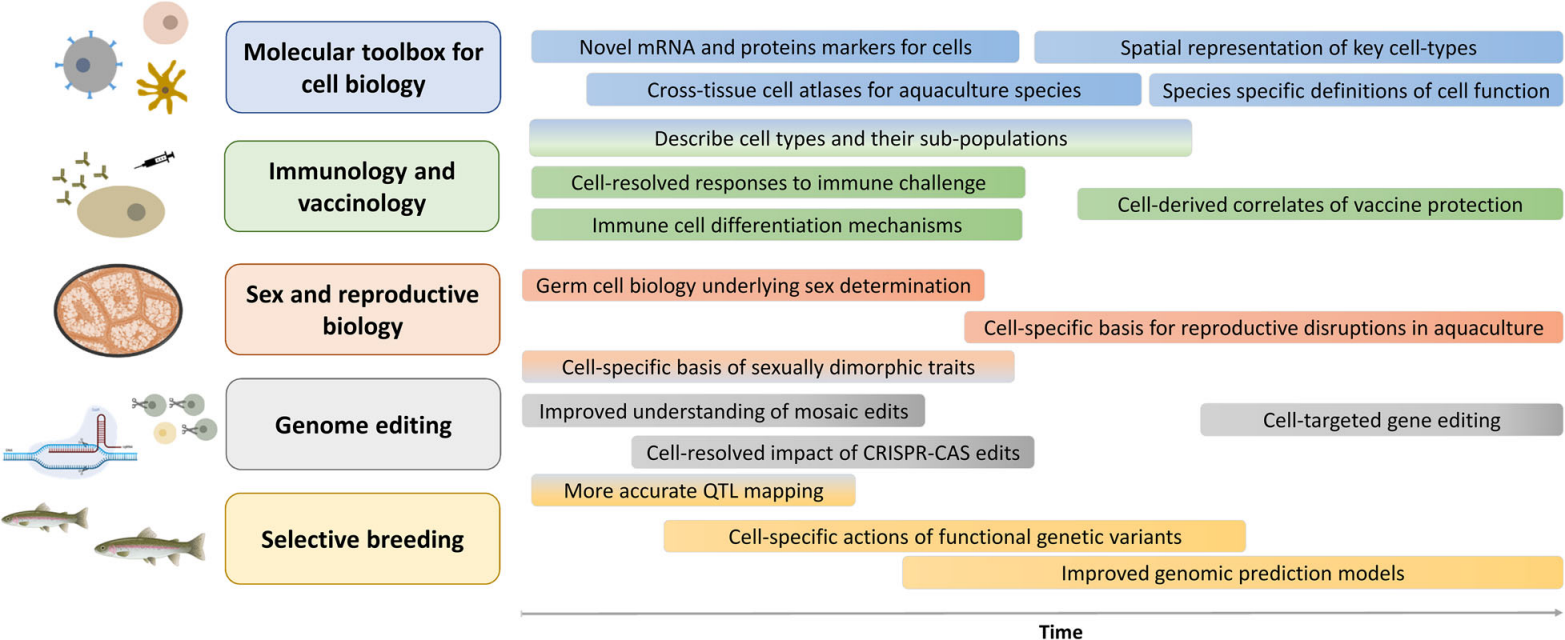
Transformative applications of single cell genomics in aquaculture research and innovation. On the left side of the figure, we outline areas (boxes with emboldened font) where uptake of single cell sequencing technologies can lead to major advances. Moving from left to right, the text boxes summarize advances we expect to arise through time, in each specific area.

The majority of knowledge on animal cell biology derives from a small group of well‐characterized organisms that benefit from advanced research ‘toolboxes’, allowing known cell types to be routinely isolated and manipulated (e.g., Ref. 47). Farmed fish and shellfish, by contrast, are a highly diverse group of >500 species^48^ presenting enormous variation in cellular biology and tissue organization, yet have limited species‐specific research tools available to target or enrich particular cell types (e.g., monoclonal antibodies and marker genes for well‐characterized cells). Consequently, our understanding of cell biology in aquaculture species remains in its infancy compared to the most characterized animal species. The uptake of single cell genomics, which allows for the unbiased high‐throughput characterization of single cells, offers an unprecedented and immediate opportunity to fast‐track our understanding and exploitation of cell biology across the great diversity of aquaculture species (Figure 1; Table 1). This section is not intended to be exhaustive of all applications of single cell transcriptomics in aquaculture research and innovation expected to arise in the coming years. Instead, our goal is to provide some illustrative directions in which single cell transcriptomics will advance different fields, which can be built up in the future.

### 3.1 Up‐step in fundamental cellular biology and molecular toolboxes

In aquaculture species, major knowledge advancement is possible using single cell transcriptomics for the identification of cell types and their expression profiles/responses (Table 1). Such work can reveal which cell types and their sub‐populations are conserved with a reference species (e.g., model organism), generate evidence for novel cell types, and resolve which cells are the likely drivers for a shift in phenotypic status. Single cell transcriptomics creates an abundance of novel marker genes for cell types and their sub‐populations, which can be used to add cell‐specific resolution to bulk gene expression studies (i.e., using existing or new datasets), while enhancing the molecular toolbox for aquaculture research. This can be as straightforward as identifying candidate gene markers for a cell population of interest, and quantifying the expression of such genes in bulk samples using targeted assays (e.g., quantitative PCR), providing a routine and cost‐effective read‐out on a cell population's phenotypic status. Cell‐specific marker genes can be taken forward as targets for in situ expression analyses, to validate and provide spatial resolution to their expression (or co‐expression with other marker genes) at the tissue level of organization (e.g., Refs. 49, 50). A subset of genes will represent targets for which to develop monoclonal antibodies targeting candidate cell surface markers, which can subsequently be used to sort or quantify cells using fluorescence‐activated cell sorting (FACS); particularly useful in immunology (Section 3.2) (reviewed in Ref. 51). Finally, data generated by single cell transcriptomics can be used to deconvolute cell‐specific expression signals in bulk studies (e.g., Refs., 52, 53, 54). This approach may provide the benefits of single cell resolution in larger functional genomics studies, adding significant value without adding additional sequencing costs. For example, a bulk RNA‐seq experiment with a complex design could be performed, which is cost‐effective per sample, and followed by deconvolution methods that leverage existing single cell transcriptomic data from the same tissue type, with the only additional costs being for the data analysis.

Considering the current status for model species alongside similar aspirations for livestock (e.g., under the FAANG initiative^55^), many commercially important aquaculture species will likely soon (i.e., in the next 5 years) benefit from cell atlases spanning different tissues relevant to production and health, mirroring what has been done, albeit at a smaller scale, in mice,^56^ zebrafish,^57^ humans^58^ and *Caenorhabditis elegans*.^59^ Such efforts have been merged and integrated into curated resources available for wider exploitation (e.g., Ref. 60), providing a template for aquaculture research communities in the future.

### 3.2 Immunology and vaccinology

The outcome of infectious disease challenge, and the success of vaccination programmes for species with an adaptive immune system, is largely a product of immune cell actions and the responses of these cells to pathogen signals, involving immune cell activation and differentiation, in addition to cell‐to‐cell interactions. Single cell transcriptomics provides a wealth of information on such processes,^61^, ^62^ enabling a new frontier of investigations into the immune system of aquaculture species.

Unsurprisingly, immunology has been a primary focus in single cell transcriptomics studies published to date in aquaculture species (e.g., Refs. 17, 23, 24, 25, 26, 30, 31, 63, 64; Table 1). Such work has identified novel heterogeneity in the haemocytes (immune cells responsible for phagocytosis in the haemolymph) of kuruma shrimp,^31^ white shrimp,^65^ and oysters.^32^ For example, a recent snRNA‐seq study in white shrimp provided evidence for phagocytic haemocytes sharing marker genes with vertebrate macrophages, offering novel future avenues to exploit the basis for cellular immunity in crustaceans.^65^ The work done to date has also demonstrated that farmed teleosts from different families (Salmonidae, Cichlidae and Serranidae) possess diverse immune cell types with identifiable subsets, including T and B lymphocytes, granulocytes, macrophages and dendritic cells (Table 1). A scRNA‐seq study focussing on circulating B cells in rainbow trout provided evidence for extensive B cell heterogeneity, likely representing distinct maturation and differentiation states, while also noting substantial differences in B cell marker genes with mammals.^25^ Two recent multi‐organ scRNA‐seq studies in turbot provided a major step forward in immunology for this species, demonstrating extensive diversity in multiple immune cell subtypes, along with associated marker genes.^64^, ^66^ This work evidenced the complex role that T cell heterogeneity plays in the response of turbot to bacterial infection,^64^ alongside evidence that neutrophils play a central role in turbot trained immunity,^66^ a process where the innate immune system is more effective in responding to a pathogen due to previous exposure to immunological stimuli. This latter finding could support the design of approaches that stimulate the innate immune system to increase disease resistance independent of vaccination.

Our own study of Atlantic salmon liver used snRNA‐seq to uncover the crucial role played by hepatocyte state in the early immune response to bacterial infection, supported by cell‐specific responses of hepatic immune cell sub‐populations.^24^ We identified a dominant population of hepatocytes that dramatically remodelled its transcriptome following infection ‐ repressing metabolic and anabolic pathways, while activating the host defence response and up‐regulating key genes controlling protein synthesis and secretion, presumed to support the translation and secretion of high concentrations of acute phase proteins into circulation.^24^ An snRNA‐seq study of orange spotted grouper brain following challenge with nervous necrosis virus (causative agent of viral nervous necrosis in many marine teleosts), revealed heterogeneity in brain macrophages, and described putative macrophage differentiation pathways supporting antiviral responses.^27^ This study employed a bioinformatic tool called Monocle, which aims to identify how far a cell‐type has transitioned along a developmental or differentiation state.^67^, ^68^ Monocle is one of several so‐called trajectory inference methods,^69^ which may have applicability to identify pathways of immune cell activation and differentiation in aquaculture species. For instance, a recent scRNA‐seq study of Atlantic cod spleen used an alternative trajectory inference method to reveal a potential B cell differentiation pathway leading to antibody‐producing plasma cells.^20^

Single cell transcriptomics has great potential to improve our understanding of vaccine responses in finfish, and for identifying novel correlates of protection that may expedite tests of vaccine efficacy. This represents an important opportunity, considering: (i) that the cellular basis for immunological memory in fishes remains poorly defined,^70^, ^71^ (ii) that reliable correlates of protective immunity are yet to be established for many aquaculture vaccines^72^ and (iii) the pressing need to reduce the number of fish used in vaccine testing.^73^ Single cell work in mammals (reviewed in Ref. 62) offers a useful direction of travel for farmed finfish. A recent study focussed on vaccination responses to dengue virus (DENV), known, like many viruses, to depend on T cell reactions.^74^ The authors identified a novel population of CD8^+^ T cells activated in response to vaccination with high memory/effector potential that endured for 4 months post‐vaccination and likely underpinned durable protection. These cells showed a distinct transcriptional programme dominated by metabolic genes, which proved to be specific markers identifiable from 14 days post‐vaccination.^74^ Another single cell transcriptomic study provided evidence for individual variation in vaccination response to hepatitis B virus within a human cohort, which correlated with the proportion of two rare dendritic cell populations showing distinct and highly specific marker genes (*NDRG2* and *CDKN1*). The authors showed it was possible to identify these dendritic cell subtypes by quantitative PCR of *NDRG2* and *CDKN1*, providing avenues to predict vaccine responsiveness prior to vaccination.^75^ Such studies demonstrate promise not simply to identify cellular mechanisms leading to vaccine protection, but also to identify marker genes for cell types that correlate with variation in vaccine protection outcomes across individuals, which may be present either before, or early post‐vaccination, and can potentially be measured cheaply.

### 3.3 Host‐pathogen interactions

Another emerging single cell approach with potential applications in aquaculture research involves the profiling of host‐pathogen interactions. Such methods apply sc/snRNA‐seq to samples including both a host species and an infecting pathogen or parasite.^76^, ^77^ The bulk equivalent, often called dual‐RNA‐seq, has been used to investigate problematic host‐pathogen interactions in aquaculture. This includes the joint profiling of transcriptomic responses of Atlantic salmon tissues with parasites and pathogens during infection scenarios, including salmon louse (*Lepeophtheirus salmonis*),^78^ *Neoparamoeba perurans* (causative of amoebic gill disease)^79^ and the intracellular bacterium *Piscirickettsia salmonis* (causative of piscirickettsiosis).^80^ These bulk studies have revealed genes potentially involved in host resistance, and candidate mechanisms by which parasites and pathogens circumvent host defence or otherwise interact with the host during infection. However, dual‐RNA‐seq methods cannot directly inform on cell types involved in host‐parasite interactions.

Single cell Dual‐seq (scDual‐seq) aims to directly measure cell‐specific responses and cell‐to‐cell interactions in samples containing both host and pathogens. For intracellular pathogens, such insights extend to distinguishing infected from uninfected host cells, which has proven fruitful in studies of human pathogens.^76^, ^81^ A recent study developed a bioinformatics approach to capture pathogenic viruses in infected host cells using scRNA‐seq, allowing the immune responses of infected and uninfected bystander cells to be distinguished.^82^ A similar strategy was used in an Atlantic salmon head kidney cell line to study the transcriptome of cells infected with infectious salmon anaemia virus, and compare their responses to bystander cells, providing novel insights into the interaction between the virus and host cells.^83^ As intracellular bacterial and viral infections pose a ubiquitous challenge in aquaculture (e.g., Refs. 72, 84), an improved understanding of which cell types are infected, along with individual variation in cell‐specific responses to infection, will have value when designing vaccines that aim to elicit cell‐mediated immunity, but also for elucidating cellular mechanisms underlying the genetic basis for disease resistance, for example, targets for viral entry into host cells. However, it must be noted that in some settings, co‐profiling of host and pathogen RNA brings challenges using bulk samples, and achieving effective scDual‐seq pipelines will be even more difficult.^77^

### 3.4 Genome editing

The discovery of CRISPR‐Cas systems has greatly facilitated the field of genome editing, revolutionising practically all fields of biology. The ability to modify the genome of aquaculture species has attracted great interest from both researchers and industry, and CRISPR/Cas9 genome editing has already been applied to target traits in farmed finfish species including Atlantic salmon, Nile tilapia, Channel catfish, and various carps (reviewed in Refs. 85, 86, 87, 88), in addition to farmed shrimp^89^ and oyster^90^ species. Legislation is rapidly evolving and genome editing seems to be gaining traction as a promising method to improve aquaculture sustainability and animal welfare.

The main biological challenge limiting applications of genome editing to improve aquaculture stocks is the identification of appropriate targets. Recently, the combination of genome editing and single‐cell transcriptomics has enabled the study of candidate gene function, even at genome‐wide resolution. In CRISPR screens, numerous genes are knocked‐out simultaneously in vitro, with most cells being edited for a single gene. If the appropriate construct has been used for editing, single‐cell transcriptomics can be used to simultaneously identify the guide RNA and determine the impact of the knock‐out of that gene on the cell transcriptome. This is commonly known as a perturbation screen, and has, for example, been used recently to knock‐out all expressed human genes (in cell lines) simultaneously to uncover the function of many uncharacterized genes on the basis of expression phenotypes in the edited cells.^91^ Several approaches including Perturb‐seq,^92^ CROP‐seq^93^ and CRISP‐seq^94^ rely on the same principle, that is, the identifying the RNA guide that edited each cell in parallel to measuring that cell's transcriptome. In addition to loss of function screens, CRISPR activation, allowing the selective up‐regulation of genes, was recently coupled to single cell transcriptomics in mouse embryonic stem cells, revealing key genes involved in transcriptional regulation.^95^ Perturbation screens have huge potential to improve our understanding of gene functions in aquaculture species, which currently rely heavily on extrapolations from model species. Nonetheless, these approaches are limited by the relevance of the in vitro model of choice for the specific trait of interest, and in this sense there is an acute need for the development of novel cell lines in aquaculture species.

As a reverse strategy, it is also possible to interrogate the impact of targeted genome edits for candidate genes by applying single cell transcriptomics. The combination of in vitro perturbation screens and in vivo characterisation of gene function using the above highlighted approaches offers a powerful new toolbox to identify target genes for the genetic improvement of aquaculture animals, prioritize causative genetic variants in regions of the genome explaining trait variation, and also to validate the potential impact of off‐target edits. Finally, single‐cell technologies may also help us better understand and tackle mosaicism, a frequent phenomenon where edited animals are a mixture of edited and non‐edited cells,^96^ representing a well‐known issue in aquaculture species (reviewed in Ref. 85). It remains unclear whether mosaicism is stochastic in nature or is biased towards certain cell lineages. Single‐cell sequencing may help us answer this question, and possibly lead to a better understanding of the molecular pathways underlying these potential biases, a necessary step towards improving the efficiency of in vivo editing in aquaculture species.

### 3.5 Sex and reproductive biology

Aquaculture is a relatively young industry and encompasses hundreds of unique species at different stages of domestication. One of the main challenges during the domestication of aquaculture species is achieving reproduction in captivity,^39^ an issue that affects even long‐farmed species such as Senegalese sole, forcing the industry to rely on wild broodstock and curtailing scope for selective breeding.^97^, ^98^ The lack of reproduction in captivity can have different underlying causes, ranging from impaired gonad maturation to ineffective or inexistent courtship. In finfishes, these processes depend on complex signalling along the hypothalamic–pituitary–gonadal axis, with specific cell‐types secreting different sex hormones that control sex differentiation and reproduction.^99^ The hormonal cascades involved in regulating reproductive processes in the many shellfish lineages used in aquaculture are equally complex and highly diverse (e.g., Refs. 100, 101).

Bulk transcriptomics lacks the resolution to detect subtle, cell‐type specific changes that may cause disruptions to reproduction in captivity.^102^, ^103^ In this sense, single‐cell technologies can help dissect the complex hormonal systems controlling reproduction at higher resolution, as done recently in the model teleost medaka,^104^ enabling the characterization of reproductive disruptions by comparing wild versus F1 individuals. Such information offers a strong base to tackle reproductive problems faced by aquaculture species. Simultaneously, understanding how reproductive cells integrate and process hormonal signals during sex differentiation will also shed light into sexual dimorphism, commonly affecting traits of commercial relevance in aquaculture species, such as growth.^105^ In this area, single cell transcriptomics has already provided insights into mechanisms underlying the expression of sexually dimorphic traits in mouse^44^, ^106^ and zig‐zag eel.^107^

Single‐cell technologies further have great potential to refine our understanding of sex determination. Aquatic species have extremely labile and consequently diverse sex determination systems. For instance, in fish, the current model of sex determination suggests a network of different interacting genetic and environmental factors, where small changes can tip the scale towards males or females, providing multiple opportunities for novel sex determination mechanisms to evolve (e.g., Ref. 108). The development of germ cells is intimately linked to sex determination in many species,^109^ and single‐cell transcriptomics can improve our understanding of this process during gonad development, identifying factors underpinning germ cell proliferation and the underlying genetic networks, as done recently in mammals (e.g., Refs. 110, 111), and avians.^112^, ^113^ For example, a recent study in zebra finch (*Taeniopygia guttata*) discovered three primordial germ cell sub‐types, representing the first evidence of heterogeneity in this cell type.^113^

With respect to work performed to date in aquaculture species, two single cell transcriptomics studies have provided insights into both germ and somatic cells in gonads. The first reported a comprehensive scRNA‐seq atlas of testis cells in orange‐spotted grouper, a protogynous hermaphrodite, revealing a candidate developmental trajectory of germ cells during spermatogenesis, providing novel markers genes at different stages of the transition from spermatogonial stem cells to mature spermatozoa.^28^ The second offered evidence for five distinct cell types in the ovary of Asian seabass (*Lates calcarifer*), a protandrous hermaphrodite, including germ cells; revealing novel oocyte marker genes, including shared marker genes with human oocytes.^44^ Such a comparative approach across different species may reveal shared factors underpinning sex determination and early sex differentiation, contributing to our understanding of the rapid evolutionary turnover of sex determining mechanisms and helping towards sex control efforts. Finally, an improved understanding of the transcriptome and development of germ cells, including the associated marker genes, may also be useful expedite progress in surrogate broodstock technologies, which have major future applications in aquaculture research and stock genetic improvement.^114^

### 3.6 Selective breeding

Selective breeding is the main route for the genetic improvement of aquaculture stocks. At the centre of these efforts are genome‐wide association studies (GWAS), which have identified quantitative trait loci (QTL, i.e., regions in the genome correlated with variation in a target phenotype, usually captured by SNP markers) for traits of interest in many aquaculture species, including growth and resistance to diverse diseases.^39^ Yet the causative genes and mutations underlying these QTL remain elusive, and selection efforts rely on neutral markers in linkage disequilibrium with causative genetic markers, which has limitations for cross‐generation and cross‐population selection using genome‐wide information (e.g., Ref. 115). Single‐cell studies can contribute towards dissecting QTL through more precise assessment of the genes co‐localizing with QTL. This could involve inferring cell‐specific expression of genes within QTL regions, or investigating cell‐type‐specific differences between individuals carrying distinct genotypes for the QTL. The power of scRNA‐seq to understand the cell‐specific nature of GWAS hits has been demonstrated in humans,^116^, ^117^ paving the way for similar studies in aquaculture species

Single‐cell technologies also allow for more precise definition of connections between molecular phenotypes (e.g., gene expression) and genetic variation.^118^ It is now known that most causative variants fall within regulatory regions of genomes (e.g., Ref. 119), making expression QTL (eQTL, i.e., genomic regions explaining individual variation in gene expression levels) increasingly to determine the genetic basis for trait variation in aquaculture populations. While initiatives such as FAANG aim to improve our understanding of non‐coding regions and eQTL in farmed animals, including aquaculture species,^55^ such work has been based on bulk methods. However, many eQTL are cell‐specific (e.g., Refs., 120, 121), highlighting an increasing need for cell‐resolved eQTL maps (e.g., Ref. 13). Furthermore, in addition to discovering the causes underlying QTL for prioritized traits in aquaculture, cell‐resolved eQTL can be fed directly into selective breeding models to prioritise functional variation (e.g., Ref. 122). It should also be noted that cell‐resolved eQTL analysis is possible following deconvolution of bulk RNA‐seq datasets.^52^, ^53^, ^54^ Therefore, as the generation of population‐scale bulk RNA‐seq is becoming increasingly affordable, it should be readily possible to design studies with aquaculture species that combine bulk data with a smaller set of single cell data for deconvolution, enabling eQTL analysis.

To summarise, up‐taking single cell transcriptomics into future research on the genetic basis for commercial trait variation will help increase the accuracy of selective breeding, leading to more efficient and resilient aquaculture stocks through genetic improvement.

## 4 EXPERIMENTAL CONSIDERATIONS

**FIGURE 2:**
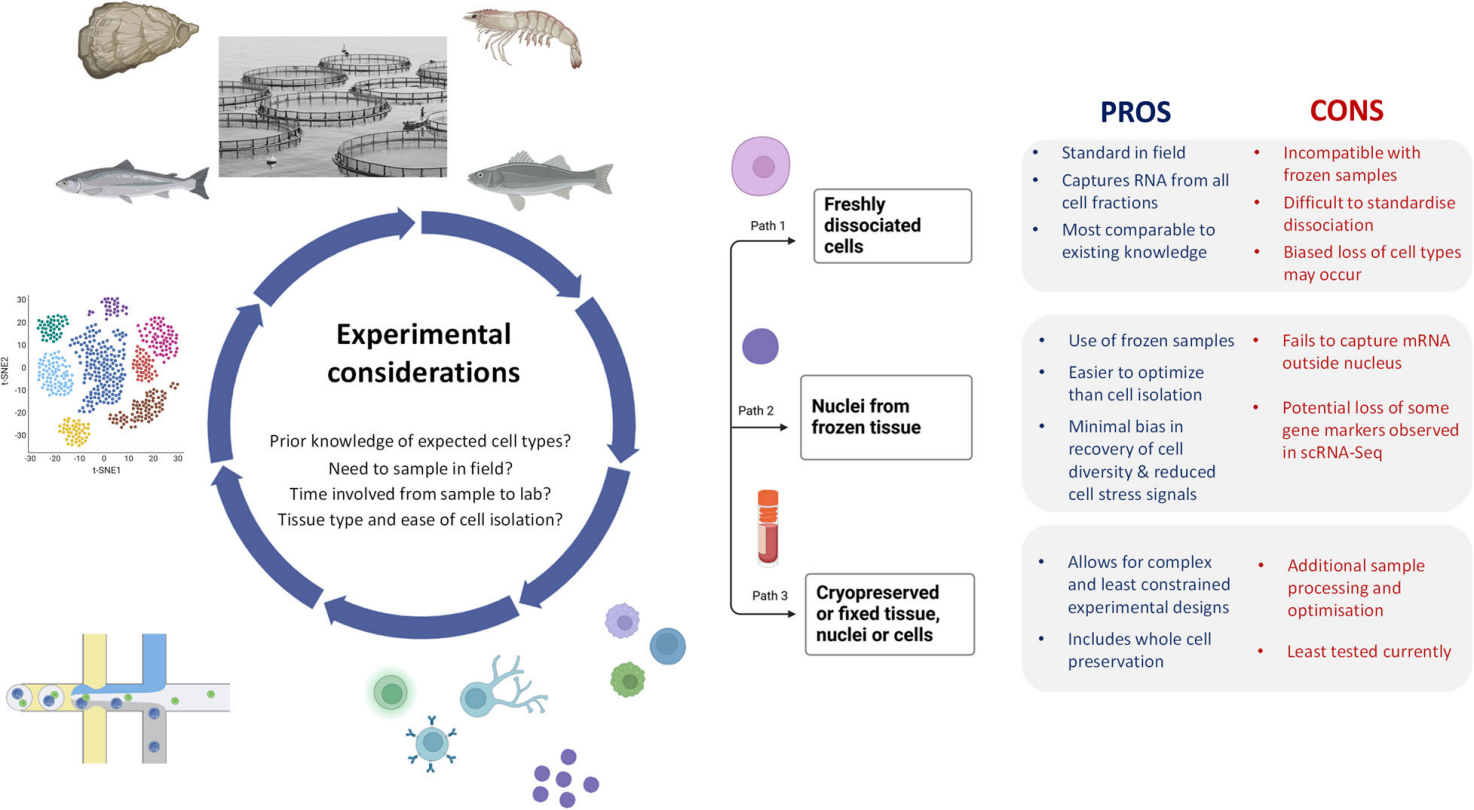
Important considerations when designing a single cell transcriptomics study, focussing on sampling and the fundamental decision surrounding whether to sequence cells or nuclei, with associated advantages and disadvantages of different approaches.

Single cell transcriptomics is performed using two fundamental strategies, scRNA‐seq or snRNA‐seq, which require cells and nuclei as the input, respectively. Consequently, a central consideration is which strategy to select (Figure 2). This decision is influenced by practical issues including whether fresh tissue is readily available, or whether it's essential to freeze samples, which may be the case when sampling aquaculture species. The quality of scRNA‐seq and snRNA‐seq data is highly correlated with the quality of cells or nuclei input to library preparation, demanding optimization efforts to ensure high‐quality outcomes are achieved. In this section, we focus on considerations when designing single cell experiments, accounting for issues faced by researchers working with aquaculture species. We first outline fundamental considerations around the choice of performing scRNA‐seq versus snRNA‐seq (Section 3.1), before reviewing methods for isolating cells and nuclei from fresh and frozen tissues as the input to scRNA‐seq (Section 4.2) and snRNA‐seq (Section 4.3), respectively.

### 4.1 Sequencing cell or nuclei transcriptomes?

A large number of studies have considered the relative merits of scRNA‐seq and snRNA‐seq across diverse biological contexts and tissues, including mammalian brain,^123^, ^124^, ^125^ kidney,^126^ liver,^127^ cardiomyocytes,^128^ adipose tissue,^129^ peripheral blood mononuclear cells, and cell lines.^125^ While the general consensus is that both strategies typically perform well using the same platform,^125^, ^130^ there are important distinctions and potential strengths and weaknesses to consider when designing an experiment. RNA captured from whole cells is derived from all compartments of the cell, which means scRNA‐seq datasets are most compatible with knowledge gained from transcriptomic studies using bulk datasets, which likewise capture all cellular fractions. Furthermore, as extensive post‐transcriptional regulation occurs outside the nucleus, scRNA‐seq and snRNA‐seq capture distinct information on gene expression dynamics, with snRNA‐seq lacking scope to capture transcriptional regulation after RNA nuclear export, but on the other hand providing more direct readouts on transcriptional regulation. Past work has shown that scRNA‐seq datasets are enriched for mitochondrial and ribosomal genes, while snRNA‐seq datasets are enriched for nuclear RNAs,^130^ including long non‐coding RNAs.^35^ snRNA‐seq data also shows a markedly higher abundance of unspliced mRNA containing intronic sequences, which has implications for downstream bioinformatics (Section 5) and may lead to biases in the expression of particular genes for some sample types (e.g., Ref. 124). Despite these clear differences, a range of studies have shown that data derived from nuclear and cell transcriptomes is highly correlated in many sample types (e.g., Refs. 128, 129, 131, 132). Furthermore, at least some of the apparent differences in gene expression between the two strategies may be due to sampling (e.g., impact of freezing vs. not freezing), rather than inherent differences between nuclei and cell transcriptomes.^35^

A known consideration is that scRNA‐seq and snRNA‐seq datasets often capture a very distinct representation of cell type diversity. Numerous studies have shown that cell diversity is biased in scRNA‐seq datasets across different tissues types (e.g., Refs. 123, 127, 133, 134), through there also exist cases where specific cell types were underrepresented in snRNA‐seq datasets (e.g., human microglia^124^). For scRNA‐seq, this issue relates mainly to the distinct sensitivity of different cell types during cell dissociation from tissues (Section 4.2), which is not straightforward for many sample types, and in some cases, it may be difficult to recover some cell types for sequencing (e.g., Ref. 135). While less well recognized, different cell types likely have distinct sensitivities to being processed through platforms used for scRNA‐seq, particularly droplet based methods employing microfluidics (Section 2). The need to dissociate cells before scRNA‐seq also activates stress associated genes (Section 4.2), which may impact downstream data interpretation (e.g., Ref. 133). This issue is thought to be largely avoided in snRNA‐seq experiments,^125^ and it is also true that nuclear isolation is more straightforward for many samples poorly amenable to cellular isolation (Section 4.3).

Building on these general issues, it is important to consider practicalities surrounding sampling using aquaculture species, which often necessitates trips to field situations (e.g., fish or shellfish farms) and/or sampling sites physically located far from the facilities where single cell transcriptomics is performed. In such cases, another challenge of using scRNA‐seq is the need to move from cell isolation to library preparation quickly to limit negative impacts on cell viability and/or cell type representation. This issue is especially important considering that we will often start with limited expectations about cell diversity/representation in samples for many aquaculture species and their tissues, making it impossible to detect biases owing to a lack of baseline understanding. On the other hand, snRNA‐seq is commonly performed using flash frozen tissue samples, which is compatible with sampling set‐ups used widely in aquaculture research. Based on the literature reviewed above, snRNA‐Seq is likely to give a less biased representation of cell types for many sample types, which is desirable when working with poorly characterized species and cells. Consequently, the recognized benefits of snRNA‐Seq are particularly relevant to studies of aquaculture species cell transcriptomes. When working with a new species or sample, it would be wise to initially compare the results of both snRNA‐Seq and scRNA‐Seq, to determine the comparative representation of cell type diversity as a trade off against the relative quality of data captured. This approach is becoming increasingly common in the mammalian literature.

### 4.2 Obtaining high‐quality cells for scRNA‐seq

High quality scRNA‐seq data are dependent on achieving cell suspensions with a high proportion (i.e., >90%) of viable individual cells. High cell viability also helps ensure that a sample's cell diversity is present at the start of library preparation. Using cell suspensions containing dead or damaged cells increases the detection of RNA located outside cells, increasing background noise in the dataset (Section 5). Achieving a high quality single cell suspension is strongly dependent on the protocol used. Generally speaking, cell dissociation involves converting fresh tissue into a heterogeneous soup of its constituent cells. It is common to dissociate tissues using mechanical means like douncing, cutting or pipetting up and down, with care required to avoid negative impacts on fragile cell types.^136^, ^137^ Enzymatic digestion is also widely used, requiring considerations around which enzyme or enzyme cocktail to employ (e.g., trypsin, collagenase, etc.), in addition to the length and temperature of digestion (typically 30–60 min at 30–37°C). It is also common to combine mechanical and enzymatic digestion.^138^ Ideally, every study will achieve a balance between releasing ‘difficult to dissociate’ cell types, while avoiding damage to more fragile cells,^35^, ^137^, ^139^ which may not be easily achieved.

Most cell dissociation protocols were developed and optimized using mammalian samples. This poses issues when working with aquaculture species, as the architecture of tissues and sensitivity of cells to mechanical or enzymatic digestion may differ greatly from mammals. It is well‐established that mammalian cells experience transcriptome‐wide changes in response to common dissociation protocols,^140^, ^141^ with incubations at 37°C inducing stress response genes.^35^ The same responses will be strongly amplified for cells from ectothermic species used in aquaculture. Providing an overview on this issue, Machado et al.^142^ concluded that virtually all cell types will express stress signatures given sufficient dissociation time. The choice of enzyme can also affect gene expression in cells.^139^, ^143^, ^144^ One possible solution to reduce negative impacts of cell dissociation is to use cold active proteases (active at <6°C), limiting cells heat stress (^142^, ^145^, ^146^). At this temperature, transcription is largely inactive in mammals, limiting artefacts linked to heat stress. This approach has been used with success in mammalian kidney,^145^ brain^124^ and solid tumour^146^ samples, and will presumably greatly reduce dissociation induced artefacts in ectothermic species.

Another way to avoid dissociation issues in aquaculture species is via the use of cells that do not require dissociation, including immune cells in the blood of rainbow trout,^25^ or oyster haemolymph.^32^ A gentle approach for cell dissociation may further be possible for soft tissues lacking extensive structure, for example, the spleen^19^, ^20^ and head kidney^21^ of teleosts. In these studies, tissues were subjected to filtering and centrifugation to achieve cellular dissociation, which can be carried out at 4°C to limit stress responses. In summary, diverse options are available for cell dissociation, but care must be taken to limit the negative impacts of enzymatic digestion and associated heat stress. Ideally, every new tissue type will be subjected to trials to achieve optimal viability of cells before scRNA‐seq.

The need to rapidly process fresh samples for library preparation in scRNA‐seq can also be circumvented using fixation protocols. These include the use of fixatives including methanol^147^ and formaldehyde,^46^ in addition to cryopreservation in DMSO.^148^ Such options provide more flexibility when sampling and storing samples for scRNA‐seq, but will still result in the same dissociation biases associated with using fresh cells. A promising method called ACME (ACetic‐MEthanol) dissociation was recently established that overcomes this issue by simultaneously dissociating and fixing cells for later sequencing.^149^ Here the authors demonstrated that scRNA‐seq after ACME dissociation avoided biases in cell diversity, emphasising benefits of maintaining the complete cell transcriptome—in other words, avoiding limitations of scRNA‐seq, while allowing cells to be stored and sequenced at a later date, that is, a major benefit of snRNA‐seq.

### 4.3 Obtaining high quality nuclei for snRNA‐Seq

For the reasons outlined in Section 4.1, it may not be possible or desirable to dissociate cells from fresh tissue samples, especially when working with aquaculture species. In such cases, flash freezing freshly‐sampled tissues on liquid nitrogen or dry ice is compatible with the recovery of high quality nuclei for snRNA‐seq at a later date, following a period of storage at an ultra‐low temperature. An important consideration, as with any bulk experiment, is that the integrity of RNA will degrade through time even at very low temperatures. Therefore, it is advantageous to minimize the time samples are frozen prior to nuclear isolation and snRNA‐seq library preparation, though it is not possible to give precise guidance here, given that there will be considerable variation in RNA degradation rates across tissues and species. Additional advantages of snRNA‐seq include that standardising nuclear isolation across different tissue types is less onerous than attempting the equivalent cell dissociations, and that nuclear isolation protocols are done on ice at 4°C, limiting the transcriptional activity of nuclei and associated impacts on gene expression.^35^

Many of the first snRNA‐seq studies employed a nuclear isolation protocol using a standard commercial nuclear isolation buffer (‘EZprep’) with a combination of douncing and centrifugation.^132^ This protocol has been modified to incorporate a sucrose gradient to accommodate more delicate tissue types.^150^ However, a drawback when using a sucrose gradient is this additional step increases the time between dissociation and library construction, potentially damaging or losing fragile nuclei. A similar protocol has been used in other sequencing assays that require nuclei from frozen tissue, including ATAC‐seq.^151^ With minor adjustments such as swapping the protease inhibitor cocktail for RNAse inhibitor, these protocols can be readily adapted for snRNA‐seq.

A growing volume of literature has compared methods of nuclear isolation across different tissues.^13^, ^36^, ^152^ This work highlights disadvantages in the original EZprep method, including nuclei loss and high levels of ambient RNA. The faster and cheaper chopping extraction approach^152^ was shown to represent the most effective method for nuclear isolation with frozen tissue in terms of capturing diverse cell types and reducing background RNA.^13^, ^36^ Chopping extraction is when nuclei are dissociated from cells using a custom nuclear extraction buffer, while chopping with precision scissors. In Eraslan et al.^13^ a toolbox is presented to optimise detergent use for chopping extraction in different tissue types that is very applicable to different species. With minor modifications, these protocols have been used in a diverse range of tissue panels in Atlantic salmon for successful snRNA‐seq (e.g., Ref. 24). The addition of RNAse to this protocol is desirable to reduce RNA degradation and background ambient RNA, especially for tissue types that show high endogenous RNAase activity.^153^

## 5 BIOINFORMATIC AND ANALYSIS CONSIDERATIONS

The analysis of single cell transcriptomics data is more complicated than bulk RNA‐seq, owing to its higher complexity and dimensionality, which often captures the expression of tens of thousands of genes in thousands of cells or nuclei. The data tends to be much sparser, consisting largely of zeros, with sequencing depth varying extensively between different cell types. These features require dimensionality reduction approaches to make the analysis computationally tractable, alongside statistical methods that compensate for sparseness and noise in the data, in addition to inventive visualisations that make the outputs interpretable. Reviews exist elsewhere that outline general approaches to single cell transcriptomic data analysis, for example, Ref. 154, and this section mainly outlines considerations relevant to studies with aquaculture species, which transfer well to other non‐model organisms (also see review by Ref. 155). The analysis pipelines discussed were designed for droplet‐based technologies being widely applied in aquaculture species (Section 2, Table 1), but can generally be used with data derived from microplate based methods like SPLiT‐seq.

### 5.1 Limitations of genome annotations and cross‐species cell markers

A key outcome of single cell transcriptomic data analysis is the generation of a count matrix, representing the number of sequencing reads or UMIs captured for each gene per cell/nuclei (the basis for all downstream analyses and visualizations). In the first step, the sequence data are usually mapped against an annotated reference genome to determine read counts for genes (Section 5.2). While analysis frameworks exist that do not require this a reference genome,^156^, ^157^ they have not been widely benchmarked.^155^ There are many considerations surrounding genome annotation that impact on data generation and interpretation. The first is that if a genome assembly is of low quality and fragmented, this will impact gene prediction, meaning key marker genes may be missing, split or only partially represented in the predicted gene models. Annotated reference genomes are available for many aquaculture species,^39^ with most being of high quality owing to modern sequencing technologies. However, even in complete and accurate reference genomes, many correctly predicted gene models will be assigned names that are challenging to interpret. This results from the fundamental nature of functional annotation (assigning names or features to genes based on sequence characteristics), which is primarily derived from similarity to gene products from characterised species in public databases. The consequence is that genes may be named incorrectly, have low‐confidence annotations, or lack functional annotations (e.g., ‘uncharacterised protein’).

An associated problem is that gene names assigned by automated genome annotation may often fail to represent the true orthologue (i.e., same gene inherited in different species from their common ancestor) to the genes from which their names were derived. Many gene families have complex evolutionary histories, characterized by losses and expansions, in addition to divergent evolutionary rates across lineages, which challenges accurate gene name assignment based solely on sequence similarity. For such gene families, sophisticated phylogenetic approaches may be required for accurate homology assignment (e.g., Ref. 158). As a simple example, if a gene has been lost by pseudogenization during the evolutionary history of a target species, it may be assigned a name for the next most closely related gene from a larger gene family. In other cases, gene families have been biasedly expanded in a particular species of interest, such that multiple co‐orthologues exist to single genes in taxa from which gene names have been derived. This represents the rule for species with a recent history of whole genome duplication (WGD), including some of the most important farmed finfishes globally; that is, salmonids^159^, ^160^ and cyprinids,^161^, ^162^ which occurred on top of a WGD event in the common teleost ancestor. In such species, it is common for there to exist three or four genes sharing equal orthology to mammalian species, and these duplicated copies often show distinct expression patterns,^160^, ^162^ which presumably extends to different cell types, for example, Ref. 24. However, duplicated genes retained from recent WGD events are poorly annotated in public sequence or genome databases, so care is required to ensure they have been properly captured in genomics studies to avoid spurious conclusions about functional differences between a target species and well‐characterised taxa with more compact gene families.

Such issues are common to comparative genomics studies involving non‐model organisms, but are particularly important to consider in single cell transcriptomics, owing to the standard practise of determining cell identity on the basis of cell marker genes. This process inescapably requires transfer of knowledge about cell marker genes from well‐characterised species. Lying at the heart of this strategy is an assumption of genetic orthology, which as detailed above may often not be met, in addition to the assumption that gene cell‐type expression is typically conserved across species.^163^ On this latter point, we already know from single cell studies of species with well‐annotated genomes where gene orthology assignment is straightforward (i.e., humans vs. mice), that while some orthologous genes are reliable cross‐species markers for the same cell types, others are not.^24^ Moreover, for species that possess multiple co‐orthologues of marker genes from well‐characterized species, it is clearly important to consider them holistically, rather than in isolation, to avoid spurious conclusions. For example, Taylor et al. identified that salmonid co‐orthologues of established mammalian marker genes for specific hepatic cell types showed highly distinct cell‐specific expression, pointing to the need for more work to define such patterns globally.^24^

Despite the above issues, single cell studies of aquaculture species cited elsewhere in this review indicate that a sufficient number of conserved marker genes exist to confidently identify major cell types, for instance, the main classes of immune cells shared by all jawed vertebrates (i.e., B cells, T cells, macrophages, dendritic cells, etc.) in recent teleost work. Where things get more challenging is in assigning identity to distinct subsets within conserved cell types, which may have evolved recently, reducing the effectiveness of marker gene information from distantly related species. A good example is the extensive heterogeneity observed in rainbow trout B cells, with distinct subsets identified lacking shared marker genes for B cell subsets in mammals.^26^ As a separate related point affecting our ability to transfer knowledge on cell markers across species, technical differences between studies, such as the use of snRNA‐seq versus scRNA‐seq, as well as the platform used for analysis, can also change the repertoire of captured marker genes, even for the same species (e.g., Ref. 127).

The above points are intended to highlight the need for a critical approach to cell identification in non‐model aquaculture species, including the need to be aware of the possibility of species‐specific cell biology, marker genes that have yet to be characterized and limitations in the use of marker genes from distantly related taxa. Nonetheless, several strategies exist to address such challenges and support a more reliable analysis. Firstly, if there are prioritized genes of interest, it is possible to manually annotate them before adding these to the reference genome annotation. For example, in a recent single cell study in turbot,^64^ manual annotations of novel immune‐type receptors (NITR) were added to the annotation prior to mapping using BLAST searches against the better annotated zebrafish genome. It can also be useful to bolster the quality of gene annotation using databases containing phylogeny‐derived information on homology relationships. For example, the Ensembl database, via the Biomart function,^164^ allows researchers to extract information on the predicted orthologue to their full set of genes from any species in Ensembl. We perform this approach routinely to compare annotations from salmonid genes to their predicted human, mouse and zebrafish orthologues. For taxa that are not included in Ensembl, global orthology predictions can be derived using methods such as Orthofinder,^165^ and carried into single cell data analysis and interpretation (e.g., Ref. 166). On a smaller scale, constructing manual phylogenetic trees for key gene families is a useful strategy, as done recently in Atlantic cod, revealing several questionable annotations in the reference genome.^19^ Overall, we advise that all single cell transcriptomic studies done in aquaculture species are based on a foundation that attempts to capture and interpret the correct evolutionary relationships between a target species and the taxa from which knowledge of cell biology is being inferred or transferred.

### 5.2 Data mapping and initial filtering

The first step towards generating a count matrix is to determine the genomic, cellular, and transcript origin of each sequenced read. This is typically performed using a single pipeline, which determines the genomic origin of reads via alignment to a reference genome, and assigns the cell or nuclei of origin using the cellular barcode (CB) and (when applicable) the transcript of origin by the UMI associated with each read. A popular pipeline to perform these steps is the 10x Genomics Cell Ranger software, which aligns reads to a reference genome, and associates each read to an error‐corrected CB and UMI. Alternative packages such as STARsolo^167^ or Alevin^168^ perform the same function as Cell Ranger but also allow for the adjustment of sequence alignment parameters, which has particular value when working with non‐model species, and the use of non‐10x cellular/UMI barcode configurations.

Multi‐mapping occurs when a read can be assigned to more than one location in the genome with similar statistical probability. This is a major issue for lineages with genomes characterized by the extensive presence of duplicated genes retained from recent WGD events, including salmonids and cyprinids. In some cases, duplicated genes are so similar that a significant proportion of reads map equally well to both locations in the genome. If we retain only the uniquely mapping portion of reads in species where duplicated features are common, extensive data loss (including marker genes) may occur, which will reduce the power of downstream analyses. Cell Ranger automatically discards multi‐mapping reads that map to more than one gene, whereas Alevin and STARsolo offer models allowing probabilistic assignment of multi‐mapping reads across duplicated genes. Thus, more data can be retained, and an effort is made to accurately estimate expression levels of duplicate genes using information about the number of uniquely mapping reads to the different gene copies. Alevin and STARsolo also offer more flexible approaches for error correcting CBs and UMIs, as well as allowing user‐specified read structures to allow the processing of data from non‐10x Genomics platforms. There are several other differences, notably faster running time than Cell Ranger, as well as differences in final UMI and gene counts, which are described elsewhere.^169^

The final step is to determine which CBs are associated with real cells or nuclei, based on the raw UMI count associated with each CB.^170^ This step is non‐trivial and even the best algorithms can result in the erroneous filtering of genuine low‐UMI count cells. For example, the incorrect filtering of neutrophils in mammals by Cell Ranger, due to low RNA levels and high RNAse content, is a known issue that needs manual intervention to address.^171^ Suggested solutions are to bypass the automated cell filtering step and specify a set number of cells to be returned, or alternatively count intronic reads in addition to exonic reads to increase UMI count (both of these approaches are possible in all tools). This issue is likely to be particularly important for aquaculture species due to the current sparsity of studies profiling cells in these species, and the heterogeneous nature of datasets generated from whole tissues. For instance, in Atlantic salmon, there can be an order of magnitude difference in the RNA content of cells (e.g., Ref. 23) and erythrocytes in particular have extremely low RNA content and can be erroneously filtered by automated tools.^24^

### 5.3 Additional filtering and quality control steps

After the count matrix has been generated, there are a number of additional steps that can be performed to enhance quality of downstream data. Most commonly, this involves a manual inspection of data to remove empty droplets and poor quality cells, the use of bioinformatic tools to remove ambient RNA, the removal of ‘doublets’ (i.e., chimeric transcriptomes derived from more than one cell or nuclei), and imputation of missing expression values. The removal of empty droplets or poor quality cells is conducted through filtering thresholds on UMI count, gene count and mitochondrial content, and is usually conducted in downstream packages such as Seurat^172^ and ScanPy,^173^ both of which include detailed vignettes on the process. These packages also provide a diversity of clustering, differential expression and visualisation options and are recommended for many downstream analysis purposes such as dimensionality reduction, clustering and visualisation (Section 5.4).

In principle, each droplet in a droplet‐based single cell dataset should contain either RNA originating from a single cell or nucleus, or no RNA at all. In reality, even in high quality datasets every droplet contains non‐negligible amounts of contaminating RNA,^174^ and this can vary significantly between datasets. This effect can be readily observed in a gill snRNA‐seq dataset from Atlantic salmon^23^ and a spleen scRNA‐seq dataset from Atlantic cod,^19^ where all cell types ‘express’ haemoglobin, that has likely originated from erythrocytes. Similarly, abundant hepatocyte genes encoding acute phase proteins showed leakage to all cell types in a liver snRNA‐seq dataset in Atlantic salmon.^24^ Several effective tools including SoupX,^174^ CellBender^175^ and DecontX^176^ have been designed to remove ambient RNA, using the presence of known empty droplets containing only ambient RNA to estimate ambient RNA content in non‐empty droplets. Cellbender in particular is highly suitable for the analysis of data generated in aquaculture species, as it requires little prior knowledge of the data and few user set parameters, while SoupX and DecontX each require input on meaningful cell identifications, which may be challenging to establish in the early stage of analysis with a novel species. Cellbender has the advantage of also performing cell filtering based on the de‐contaminated dataset, when the removal of ambient RNA can clarify the distinction between empty and non‐empty droplets.

#### 5.3.1 Doublet removal

Three approaches exist to deal with doublets in single cell datasets. The simplest is to apply an upper threshold on the UMI or gene count in the quality control step, under the assumption that doublets will contain more RNA and therefore more UMIs/genes. While this may work well in homogeneous data sets where all cells/nuclei express similar number of transcripts, in a tissue level dataset there will often exist enormous variation in transcriptional activity between cell types, resulting in an upper threshold erroneously removing the most transcriptionally active cells, while missing doublets containing less active cells. Therefore, this strategy is not generally recommended.

The second approach is to cluster the cells or nuclei (e.g., with Seurat or ScanPy) (Section 5.4), perform a differential gene expression test between each cluster and all other cells, and manually identify clusters that differentially express specific markers of two other cell types, but no unique markers of their own. This approach can be time consuming, allows for human error, and only identifies heterotypic doublets (i.e., two different cell types) but has the advantage of a biological justification for the removal of cells.

The third approach is to employ a dedicated bioinformatic package, many of which have been usefully benchmarked against each other.^177^ These usually operate by generating a set of artificial doublets based on an initial clustering of the data, which are used as the basis to identify actual doublets in a sample. In our experience, care should be taken when using a doublet removal package, as there can be significant disparities between packages in which cells are identified as doublets. A manual inspection of the cells identified as doublets is recommended prior to removal. The scDblFinder package^178^ uses a function that identifies clusters of likely heterotypic doublets based on an algorithmic version of the manual strategy of performing differential gene expression tests and identifying cell clusters that express markers of two other clusters, but no unique markers.

#### 5.3.2 Imputation

Imputation aims to fill missing data values by comparison to a ‘true’ reference. In the context of single cell data, this means using transcriptionally similar cells to impute missing expression of genes that have been lost due to incomplete capture or sequencing of the RNA in the cell. The rationale for using imputation is that the imputed dataset will be superior for downstream analysis, but this is by no means clear. For example, while the recovery of gene expression profiles observed in bulk RNA‐seq can be enhanced through imputation, this may result in little downstream enhancement to clustering and trajectory analysis,^179^ and there is the potential to introduce false positive results in differential gene expression tests.^180^ Despite this, imputation has been used to recover biologically meaningful expression of very lowly expressed genes that was not present in the non‐imputed dataset (e.g., Ref. 181). Imputation should be conducted with care and the benefits weighed against the risks of introducing unwanted artefacts.

### 5.4 Clustering and cell type identification

To characterize the heterogeneity of cells in a single cell dataset, it is necessary to group cells sharing similar molecular profiles in a process known as clustering, which is fundamental to most downstream applications. Most clustering algorithms use machine learning approaches to cluster cells in an unsupervised fashion (i.e., without user input) and generally perform well.^182^, ^183^ Prior to clustering, data dimensionality is reduced to make the analysis computationally tractable and to remove uninformative variation. This typically involves using a subset of genes that show the highest level of expression variation (perhaps 5%–10% of genes), before performing further dimensionality reduction, such as principal component analysis (PCA), and selecting the PCs explaining the most variation to eliminate uninformative noise. Cells are then grouped by similarity along these axes, with a graph‐based clustering algorithm used to determine cluster number and assign cells.

Two critical parameters to consider during clustering are ‘resolution’, defining how fine‐grained the definition of cell types will be, and the degree of variation used to inform the clustering (i.e., the number of PCs used as input). A study aiming to describe only the broad cell lineages present in the data should opt for low resolution clustering, requiring few PCs, while a study attempting to describe all variation, including heterogeneity within particular cell lineages, should opt for high resolution clustering, requiring more of the variation in the dataset (hence more PCs). For most datasets, particularly in under described aquaculture species, the clustering process will inevitably require multiple attempts at clustering, with better understanding of the data from early clustering attempts leading to better informed choices for parameters. With very heterogeneous datasets, such as those derived from primary tissue, the best approach is often to perform an initial global clustering to identify the major cell types in the samples, then sub‐setting these cell lineages and performing separate clustering with parameters tuned to each lineage, for example, Ref. 24. This approach is commonly used and avoids under clustering very diverse cell lineages (e.g., haematopoietic, including immune cells) or over clustering homogeneous populations, but is time consuming. Alternatively, other systematic approaches have been developed to deal with this issue.^184^, ^185^

At this stage on an analysis, it is important to visualise the data in order to assess the performance and biological relevance of the clustering. Currently the most widely used visualisation of cell clustering is uniform manifold approximation and projection (UMAP),^186^ a nonlinear embedding method that aims to project all variation in data onto two axes, resulting in a “map” where the proximity of individual cells reflects similarity in their transcriptome composition. Identification and annotation of cell types involves the use of ‘a priori’ knowledge of marker genes to assign cellular identity. This can be performed by either visualising the expression of these markers in each cluster, for example with “violin” plots or heatmaps, or by performing differential gene expression tests between each cluster and all other cells, then referencing the most differentially expressed genes in each cluster against the ‘*a priori*’ markers. The design of differential expression tests employed to define cell marker genes is important to the outcome of this approach. For example, when investigating cell sub‐types within a lineage, the resulting marker genes from the differential expression tests strongly depends on the background used for comparison, that is, whether the test is performed against all other cells in the experiment, or against only other cells in that lineage. For example, in Atlantic salmon liver, T cell subsets were better identified by comparison of expression within the T cell lineage, rather than against all other liver cells. For this reason is it often useful to analyse subsets of the data separately when attempting to annotate cell sub‐types. Once clustering and annotation of cell types has been completed, many options exist for downstream analyses, and the choice will be informed by the goal of the study. Table 2 summarises several of the common bioinformatic analyses that can be performed.

**TABLE 2:** Summary of potential downstream bioinformatic analyses in single cell transcriptomic studies

Application	Purpose	Challenges	Example tools
Differential expression tests between conditions	Identify cell type specific changes in gene expression in response to challenge or changes in environment.	Low numbers of biological replicates may result in false positives. Low RNA content in single cell datasets may lead to bias against lowly expressed genes being accurately identified as differentially expressed.	Many bulk RNA‐seq differential gene expression tests work well with single cell data, in addition to single cell specific tests. A variety of DGE tests are available in scRNA‐seq workflows, Seurat,^172^ SCANPY^173^ and Scater.^190^
Trajectory inference	Infer dynamic changes in transcriptomic profiles, for example, along a developmental trajectory.	Sampling must take place at the timepoints where dynamic changes are taking places. Uncertainty in number of cells required to reliable infer trajectories.	Monocle^68^ Velocyto^191^ Slingshot^192^
Deconvolution of bulk RNA‐seq datasets	Estimation of cellular composition of bulk RNA‐seq data.	No aquaculture specific challenges.	scBio^193^ Bisque^194^
Gene network analysis	Identify signalling pathways and molecular interactions between cell populations.	Reliance on derived knowledge of molecular interactions, which is not available for most species Noisy nature of single cell data can confound inference of networks.	CellPhoneDb^195^ BTR^196^

## 6 FUTURE PERSPECTIVES AND CONCLUSIONS

Looking ahead, spatial ‘omics’ is a related set of technologies we expect to make a big impact on the characterisation of cell biology in aquaculture species. A limitation of the single cell methods reviewed here is the lack of in situ data to contextualize cell‐specific gene expression (including in response to stimuli) in the background of where the cells are physically located or co‐located within a tissue. The spatial organization of cells within tissues is vital to cell and tissue function, and may radically change under different physiological conditions, for example, the migration and interaction of cells of the immune system following disease challenge. Several methods are already widely used to explore gene or protein expression within cells and tissue organizations (e.g., in situ RNA hybridization and immunohistochemistry), which can be combined with novel cell marker genes gained from single cell transcriptomics. However, such approaches are limited in throughput. Spatial transcriptomics encompasses a group of recently developed methods that bridge the gap between low‐throughput in situ expression methods, and high‐throughput single cell transcriptomics (reviewed in Ref. 187). In essence, these methods capture transcriptomic read‐outs in minute regions (tens of micrometre scale) sampled from tissue sections, maintaining the spatial location of each region to build up a bigger picture of gene expression across the sampled tissue. This approach is complementary to single cell transcriptomics, helping to interpret the function of cell types according to their spatial location in relation to known features of a tissue, particularly insightful when used in an experimental framework comparing different conditions.

To wrap‐up, single cell genomics is being rapidly uptaken in aquaculture species, and when applied alongside other emerging technologies will revolutionise our understanding of the cell‐specific basis for traits of significance to sustainability and production goals. We have outlined some of the key envisaged applications and potential barriers to successfully adopting single cell technologies in aquaculture research, highlighting considerations for experimental design and execution both in the lab and during data analysis, with major implications for sampling decisions, data quality and interpretation, and even cost. All single cell studies require careful consideration and planning, and there exist no blanket options to guarantee standardized high quality data and interpretations in all species and systems. Clearly this field is moving rapidly, and will build rapidly upon the emerging knowledge gained from pioneering studies published in recent years, providing increasing assurance concerning methods best suited to different aquaculture species. In the future, we envisage a data‐derived approach will increasingly drive forward advances in species‐specific cell biology, which is needed to move beyond the current constraints of knowledge transfer from a few well‐characterised species.

## AUTHOR CONTRIBUTIONS

**Rose Ruiz Daniels:** Conceptualization; writing – original draft; writing – review and editing; visualization; funding acquisition. **Richard S. Taylor:** Conceptualization; writing – original draft; funding acquisition; writing – review and editing. **Diego Robledo:** Conceptualization; funding acquisition; writing – original draft; writing – review and editing; supervision; visualization; project administration; resources. **Daniel J. Macqueen:** Supervision; conceptualization; funding acquisition; writing – original draft; writing – review and editing; visualization; project administration; resources.

## FUNDING STATEMENT

We acknowledge funding for our single cell research, including grants from the Scottish Universities Life Sciences Alliance (Technology Seed Funding Call) (all authors), the University of Edinburgh's Data Driven Innovation Initiative (Scottish Funding Council Beacon ‘Building Back Better’ Call) (Rose Ruiz Daniels, Richard S. Taylor, Daniel J. Macqueen), the Biotechnology and Biological Sciences Research Council, including BBS/E/D/10002071 and BBS/E/D/20002174 (all authors), BB/W005859/1 (Richard S. Taylor and Daniel J. Macqueen) and BB/V009818/1 (Diego Robledo), and the Norwegian Seafood Research Fund 901631 (Diego Robledo).

## CONFLICT OF INTEREST

The authors declare no conflicts of interest.

## Data Availability

Data sharing is not applicable to this article as no new data were created or analyzed in this study.

## References

[1] Linnarsson S , Teichmann SA . Single‐cell genomics: coming of age. Genome Biol. 2016;17(1):1‐3. doi:10.1186/S13059-016-0960-X/METRICS 27160975 PMC4862185

[2] Stubbington MJT , Rozenblatt‐Rosen O , Regev A , Teichmann SA . Single‐cell transcriptomics to explore the immune system in health and disease. Science. 2017;358(6359):58‐63. doi:10.1126/science.aan6828 28983043 PMC5654495

[3] Griffiths JA , Scialdone A , Marioni JC . Using single‐cell genomics to understand developmental processes and cell fate decisions. Mol Syst Biol. 2018;14(4):e8046. doi:10.15252/msb.20178046 29661792 PMC5900446

[4] Aldridge S , Teichmann SA . Single cell transcriptomics comes of age. Nat Commun. 2020;11(1):4307. doi:10.1038/s41467-020-18158-5 32855414 PMC7453005

[5] Niemöller C , Wehrle J , Riba J , et al. Bisulfite‐free epigenomics and genomics of single cells through methylation‐sensitive restriction. Commun Biol. 2021;4(1):153. doi:10.1038/s42003-021-01661-w 33526904 PMC7851132

[6] Buenrostro JD , Wu B , Litzenburger UM , et al. Single‐cell chromatin accessibility reveals principles of regulatory variation. Nature. 2015;523(7561):486‐490. doi:10.1038/nature14590 26083756 PMC4685948

[7] Nagano T , Lubling Y , Stevens TJ , et al. Single‐cell Hi‐C reveals cell‐to‐cell variability in chromosome structure. Nature. 2013;502(7469):59‐64. doi:10.1038/nature12593 24067610 PMC3869051

[8] Schier AF . Single‐cell biology: beyond the sum of its parts. Nat Methods. 2020;17(1):17‐20. doi:10.1038/s41592-019-0693-3 31907464

[9] Schoof EM , Furtwängler B , Üresin N , et al. Quantitative single‐cell proteomics as a tool to characterize cellular hierarchies. Nat Commun. 2021;12: 3341. doi:10.1038/s41467-021-23667-y PMC8185083 34099695

[10] Kashima Y , Sakamoto Y , Kaneko K , Seki M , Suzuki Y , Suzuki A . Single‐cell sequencing techniques from individual to multiomics analyses. Exp Mol Med. 2020;52(9):1419‐1427. doi:10.1038/s12276-020-00499-2 32929221 PMC8080663

[11] Regev A , Teichmann SA , Lander ES , et al. The human cell atlas. eLife. 2017;6:e27041. doi:10.7554/eLife.27041 29206104 PMC5762154

[12] Elmentaite R , Domínguez Conde C , Yang L , Teichmann SA . Single‐cell atlases: shared and tissue‐specific cell types across human organs. Nat Rev Genet. 2022;23(7):395‐410. doi:10.1038/s41576-022-00449-w 35217821

[13] Eraslan G , Drokhlyansky E , Anand S , et al. Single‐nucleus cross‐tissue molecular reference maps toward understanding disease gene function. Science. 2022;376(6594):eabl4290. doi:10.1126/science.abl4290 35549429 PMC9383269

[14] Tabula Sapiens Consortium , Jones RC , Karkanias J , et al. The Tabula Sapiens: a multiple‐organ, single‐cell transcriptomic atlas of humans. Science. 2022;376(6594):eabl4896. doi:10.1126/science.abl4896 35549404 PMC9812260

[15] Kulkarni A , Anderson AG , Merullo DP , Konopka G . Beyond bulk: a review of single cell transcriptomics methodologies and applications. Curr Opin Biotechnol. 2019;58:129‐136. doi:10.1016/J.COPBIO.2019.03.001 30978643 PMC6710112

[16] Svensson V , Pachter L . RNA velocity: molecular kinetics from single‐cell RNA‐Seq. Mol Cell. 2018;72(1):7‐9. doi:10.1016/j.molcel.2018.09.026 30290149

[17] Chandhini S , Rejish Kumar VJ . Transcriptomics in aquaculture: current status and applications. Rev Aquac. 2019;11(4):1379‐1397. doi:10.1111/raq.12298

[18] Haque A , Engel J , Teichmann SA , Lönnberg T . A practical guide to single‐cell RNA‐sequencing for biomedical research and clinical applications. Genome Med. 2017;9(1):75. doi:10.1186/s13073-017-0467-4 28821273 PMC5561556

[19] Guslund NC , Solbakken MH , Brieuc MSO , Jentoft S , Jakobsen KS , Qiao SW . Single‐cell transcriptome profiling of immune cell repertoire of the Atlantic cod which naturally lacks the major histocompatibility class II system. Front Immunol. 2020;11:559555. doi:10.3389/fimmu.2020.559555 33154745 PMC7588623

[20] Guslund NC , Krabberød AK , Nørstebø SF , et al. Lymphocyte subsets in Atlantic cod (*Gadus morhua*) interrogated by single‐cell sequencing. Commun Biol. 2022;5(1):689. doi:10.1038/s42003-022-03645-w 35821077 PMC9276791

[21] Peuß R , Box AC , Chen S , et al. Adaptation to low parasite abundance affects immune investment and immunopathological responses of cavefish. Nat Ecol Evol. 2020;4(10):1416‐1430. doi:10.1038/s41559-020-1234-2 32690906 PMC11062081

[22] Hu M , Zheng X , Fan CM , Zheng Y . Lineage dynamics of the endosymbiotic cell type in the soft coral Xenia. Nature. 2020;582(7813):534‐538. doi:10.1038/s41586-020-2385-7 32555454 PMC7332420

[23] West AC , Mizoro Y , Wood SH , et al. Immunologic profiling of the Atlantic salmon gill by single nuclei transcriptomics. Front Immunol. 2021;12:669889. doi:10.3389/fimmu.2021.6698899 34017342 PMC8129531

[24] Taylor RS , Ruiz Daniels R , Dobie R , et al. Single cell transcriptomics of Atlantic salmon (*Salmo salar* L.) liver reveals cellular heterogeneity and immunological responses to challenge by *Aeromonas salmonicida* . Front Immunol. 2022;13:984799. doi:10.3389/fimmu.2022.984799 36091005 PMC9450062

[25] Perdiguero P , Morel E , Díaz‐Rosales P , Tafalla C . Individual B cells transcribe multiple rearranged immunoglobulin light chains in teleost fish. iScience. 2021;24(6):102615. doi:10.1016/J.ISCI.2021.102615 34142062 PMC8188548

[26] Perdiguero P , Morel E , Tafalla C . Diversity of rainbow trout blood B cells revealed by single cell RNA sequencing. Biology (Basel). 2021;10(6):511. doi:10.3390/biology10060511 34207643 PMC8227096

[27] Wang Q , Peng C , Yang M , et al. Single‐cell RNA‐seq landscape midbrain cell responses to red spotted grouper nervous necrosis virus infection. PLoS Pathog. 2021;17(6):e1009665. doi:10.1371/journal.ppat.1009665 34185811 PMC8241073

[28] Wu X , Yang Y , Zhong C , et al. Single‐cell atlas of adult testis in protogynous hermaphroditic orange‐spotted grouper, *Epinephelus coioides* . Int J Mol Sci. 2021;22(22):12607. doi:10.3390/ijms222212607 34830486 PMC8618070

[29] Wu L , Gao A , Li L , Chen J , Li J , Ye J . A single‐cell transcriptome profiling of anterior kidney leukocytes from Nile tilapia (*Oreochromis niloticus*). Front Immunol. 2021;12:783196. doi:10.3389/fimmu.2021.783196 35027916 PMC8750066

[30] Niu J , Huang Y , Liu X , et al. Single‐cell RNA‐seq reveals different subsets of non‐specific cytotoxic cells in teleost. Genomics. 2020;112(6):5170‐5179. doi:10.1016/j.ygeno.2020.09.031 32971213

[31] Koiwai K , Koyama T , Tsuda S , et al. Single‐cell RNA‐seq analysis reveals penaeid shrimp hemocyte subpopulations and cell differentiation process. Elife. 2021;10:e66954. doi:10.7554/eLife.66954 34132195 PMC8266392

[32] Meng J , Wang WX . Highly sensitive and specific responses of oyster hemocytes to copper exposure: single‐cell transcriptomic analysis of different cell populations. Environ Sci Technol. 2022;56(4):2497‐2510. doi:10.1021/acs.est.1c07510 35107992

[33] Naylor RL , Hardy RW , Buschmann AH , et al. A 20‐year retrospective review of global aquaculture. Nature. 2021;591(7851):551‐563. doi:10.1038/s41586-021-03308-6 33762770

[34] Stentiford GD , Sritunyalucksana K , Flegel TW , et al. New paradigms to help solve the global aquaculture disease crisis. PLoS Pathog. 2017;13(2):e1006160. doi:10.1371/journal.ppat.1006160 28152043 PMC5289612

[35] Denisenko E , Guo BB , Jones M , et al. Systematic assessment of tissue dissociation and storage biases in single‐cell and single‐nucleus RNA‐seq workflows. Genome Biol. 2020;21(1):130. doi:10.1186/s13059-020-02048-6 32487174 PMC7265231

[36] Slyper M , Porter CBM , Ashenberg O , et al. A single‐cell and single‐nucleus RNA‐Seq toolbox for fresh and frozen human tumors. Nat Med. 2020;26(5):792‐802. doi:10.1038/s41591-020-0844-1 32405060 PMC7220853

[37] Lähnemann D , Köster J , Szczurek E , et al. Eleven grand challenges in single‐cell data science. Genome Biol. 2020;21(1):31. doi:10.1186/s13059-020-1926-6 32033589 PMC7007675

[38] Nayak R , Hasija Y . A hitchhiker's guide to single‐cell transcriptomics and data analysis pipelines. Genomics. 2021;113(2):606‐619. doi:10.1016/j.ygeno.2021.01.007 33485955

[39] Houston RD , Bean TP , Macqueen DJ , et al. Harnessing genomics to fast‐track genetic improvement in aquaculture. Nat Rev Genet. 2020;21(7):389‐409. doi:10.1038/s41576-020-0227-y 32300217

[40] Chen G , Ning B , Shi T . Single‐cell RNA‐Seq technologies and related computational Data analysis. Front Genet. 2019;10:317. doi:10.3389/fgene.2019.00317 31024627 PMC6460256

[41] Zhang X , Li T , Liu F , et al. Comparative analysis of droplet‐based ultra‐high‐throughput single‐cell RNA‐Seq systems. Mol Cell. 2019;73(1):130‐142.e5. doi:10.1016/j.molcel.2018.10.020 30472192

[42] Macosko EZ , Basu A , Satija R , et al. Highly parallel genome‐wide expression profiling of individual cells using nanoliter droplets. Cell. 2015;161(5):1202‐1214. doi:10.1016/j.cell.2015.05.002 26000488 PMC4481139

[43] Klein AM , Mazutis L , Akartuna I , et al. Droplet barcoding for single‐cell transcriptomics applied to embryonic stem cells. Cell. 2015;161(5):1187‐1201. doi:10.1016/j.cell.2015.04.044 26000487 PMC4441768

[44] Liu X , Li W , Yang Y , et al. Transcriptome profiling of the ovarian cells at the single‐cell resolution in adult Asian seabass. Front Cell Dev Biol. 2021;9:647892. doi:10.3389/fcell.2021.647892 33855024 PMC8039529

[45] Picelli S , Faridani OR , Björklund AK , Winberg G , Sagasser S , Sandberg R . Full‐length RNA‐seq from single cells using Smart‐seq2. Nat Protoc. 2014;9(1):171‐181. doi:10.1038/nprot.2014.006 24385147

[46] Rosenberg AB , Roco CM , Muscat RA , et al. Single‐cell profiling of the developing mouse brain and spinal cord with split‐pool barcoding. Science. 2018;360(6385):176‐182. doi:10.1126/science.aam8999 29545511 PMC7643870

[47] Eisenhoffer GT , Slattum G , Ruiz OE , et al. A toolbox to study epidermal cell types in zebrafish. J Cell Sci. 2017;130(1):269‐277. doi:10.1242/jcs.184341 27149923 PMC5394773

[48] The State of World Fisheries . FAO. Food and Agriculture Organization of the United Nations. 2022. 10.4060/cc0461en.

[49] Strell C , Hilscher MM , Laxman N , et al. Placing RNA in context and space ‐ methods for spatially resolved transcriptomics. FEBS J. 2019;286(8):1468‐1481. doi:10.1111/febs.14435 29542254

[50] Asp M , Bergenstråhle J , Lundeberg J . Spatially resolved transcriptomes‐next generation tools for tissue exploration. Bioessays. 2020;42(10):e1900221. doi:10.1002/bies.201900221 32363691

[51] Fei C , Nie L , Zhang J , Chen J . Potential applications of fluorescence‐activated cell sorting (FACS) and droplet‐based microfluidics in promoting the discovery of specific antibodies for characterizations of fish immune cells. Front Immunol. 2021;12:771231. doi:10.3389/fimmu.2021.771231 34868030 PMC8635192

[52] Newman AM , Liu CL , Green MR , et al. Robust enumeration of cell subsets from tissue expression profiles. Nat Methods. 2015;12(5):453‐457. doi:10.1038/nmeth.3337 25822800 PMC4739640

[53] Avila Cobos F , Alquicira‐Hernandez J , Powell JE , Mestdagh P , De Preter K . Benchmarking of cell type deconvolution pipelines for transcriptomics data. Nat Commun. 2020;11(1):5650. doi:10.1038/s41467-020-19015-1 33159064 PMC7648640

[54] Jin H , Liu Z . A benchmark for RNA‐seq deconvolution analysis under dynamic testing environments. Genome Biol. 2021;22(1):102. doi:10.1186/s13059-021-02290-6 33845875 PMC8042713

[55] Clark EL , Archibald AL , Daetwyler HD , et al. From FAANG to fork: application of highly annotated genomes to improve farmed animal production. Genome Biol. 2020;21(1):285. doi:10.1186/s13059-020-02197-8 33234160 PMC7686664

[56] Tabula Muris Consortium , Overall Coordination , Logistical Coordination , et al. Single‐cell transcriptomics of 20 mouse organs creates a Tabula Muris. Nature. 2018;562(7727):367‐372. doi:10.1038/s41586-018-0590-4 30283141 PMC6642641

[57] Farnsworth DR , Saunders LM , Miller AC . A single‐cell transcriptome atlas for zebrafish development. Dev Biol. 2020;459(2):100‐108. doi:10.1016/j.ydbio.2019.11.008 31782996 PMC7080588

[58] Lindeboom RGH , Regev A , Teichmann SA . Towards a human cell atlas: taking notes from the past. Trends Genet. 2021;37(7):625‐630. doi:10.1016/j.tig.2021.03.007 33879355

[59] Packer JS , Zhu Q , Huynh C , et al. A lineage‐resolved molecular atlas of C. elegans embryogenesis at single‐cell resolution. Science. 2019;365(6459):eaax1971. doi:10.1126/science.aax1971 31488706 PMC7428862

[60] Zhang X , Lan Y , Xu J , et al. CellMarker: a manually curated resource of cell markers in human and mouse. Nucleic Acids Res. 2019;47(D1):D721‐D728. doi:10.1093/nar/gky900 30289549 PMC6323899

[61] Papalexi E , Satija R . Single‐cell RNA sequencing to explore immune cell heterogeneity. Nat Rev Immunol. 2018;18(1):35‐45. doi:10.1038/nri.2017.76 28787399

[62] Noé A , Cargill TN , Nielsen CM , Russell AJC , Barnes E . The application of single‐cell RNA sequencing in vaccinology. J Immunol Res. 2020;2020:8624963. doi:10.1155/2020/8624963 32802896 PMC7411487

[63] Chan JTH , Kadri S , Köllner B , Rebl A , Korytář T . RNA‐seq of single fish cells ‐ seeking out the leukocytes mediating immunity in teleost fishes. Front Immunol. 2022;13:798712. doi:10.3389/fimmu.2022.798712 35140719 PMC8818700

[64] Chen W , Huang J , Wang W , et al. Multi‐tissue scRNA‐seq reveals immune cell landscape of turbot (*Scophthalmus maximus*). Fundam Res. 2022;2(4):550‐561. doi:10.1016/j.fmre.2021.12.015 PMC11197760 38933994

[65] Yang P , Chen Y , Huang Z , et al. Single‐cell RNA sequencing analysis of shrimp immune cells identifies macrophage‐like phagocytes. Elife. 2022;11:e80127. doi:10.7554/eLife.80127 36200862 PMC9584607

[66] Mu D , Yang J , Jiang Y , et al. Single‐cell transcriptomic analysis reveals neutrophil as orchestrator during β‐glucan–induced trained immunity in a teleost fish. J Immunol. 2022;209(4):783‐795. doi:10.4049/jimmunol.2200225 35896333

[67] Trapnell C , Cacchiarelli D , Grimsby J , et al. The dynamics and regulators of cell fate decisions are revealed by pseudotemporal ordering of single cells. Nat Biotechnol. 2014;32(4):381‐386. doi:10.1038/nbt.2859 24658644 PMC4122333

[68] Cao J , Spielmann M , Qiu X , et al. The single‐cell transcriptional landscape of mammalian organogenesis. Nature. 2019;566(7745):496‐502. doi:10.1038/s41586-019-0969-x 30787437 PMC6434952

[69] Saelens W , Cannoodt R , Todorov H , Saeys Y . A comparison of single‐cell trajectory inference methods. Nat Biotechnol. 2019;37(5):547‐554. doi:10.1038/s41587-019-0071-9 30936559

[70] Yamaguchi T , Quillet E , Boudinot P , Fischer U . What could be the mechanisms of immunological memory in fish? Fish Shellfish Immunol. 2019;85:3‐8. doi:10.1016/j.fsi.2018.01.035 29410093

[71] Stosik M , Tokarz‐Deptuła B , Deptuła W . Immunological memory in teleost fish. Fish Shellfish Immunol. 2021;115:95‐103. doi:10.1016/j.fsi.2021.05.022 34058353

[72] Munang'andu HM , Salinas I , Tafalla C , Dalmo RA . Editorial: vaccines and immunostimulants for finfish. Front Immunol. 2020;11:573771. doi:10.3389/fimmu.2020.573771 33117370 PMC7553079

[73] Midtlyng PJ , Hendriksen C , Balks E , et al. Three Rs approaches in the production and quality control of fish vaccines. Biologicals. 2011;39(2):117‐128. doi:10.1016/j.biologicals.2011.02.001 21371907

[74] Waickman AT , Friberg H , Gargulak M , et al. Assessing the diversity and stability of cellular immunity generated in response to the candidate live‐attenuated dengue virus vaccine TAK‐003. Front Immunol. 2019;10:1778. doi:10.3389/fimmu.2019.01778 31417556 PMC6684763

[75] Aevermann BD , Shannon CP , Novotny M , et al. Machine learning‐based single cell and integrative analysis reveals that baseline mdc predisposition correlates with hepatitis B vaccine antibody response. Front Immunol. 2021;12:690470. doi:10.3389/fimmu.2021.690470 34777332 PMC8588842

[76] Avital G , Avraham R , Fan A , Hashimshony T , Hung DT , Yanai I . scDual‐Seq: mapping the gene regulatory program of Salmonella infection by host and pathogen single‐cell RNA‐sequencing. Genome Biol. 2017;18:200. doi:10.1186/s13059-017-1340-x 29073931 PMC5658913

[77] Westermann AJ , Vogel J . Cross‐species RNA‐seq for deciphering host‐microbe interactions. Nat Rev Genet. 2021;22(6):361‐378. doi:10.1038/s41576-021-00326-y 33597744

[78] Sutherland BJG , Covello JM , Friend SE , et al. Host–parasite transcriptomics during immunostimulant‐enhanced rejection of salmon lice (*Lepeophtheirus salmonis*) by Atlantic salmon (*Salmo salar*). Facets. 2017;2(1):477‐495. doi:10.1139/FACETS-2017-0020

[79] Botwright NA , Mohamed AR , Slinger J , Lima PC , Wynne JW . Host‐parasite interaction of Atlantic salmon (*Salmo salar*) and the ectoparasite *Neoparamoeba perurans* in amoebic gill disease. Front Immunol. 2021;12:672700. doi:10.3389/fimmu.2021.672700 34135900 PMC8202022

[80] Valenzuela‐Miranda D , Gallardo‐Escárate C . Dual RNA‐Seq uncovers metabolic amino acids dependency of the intracellular bacterium *Piscirickettsia salmonis* infecting Atlantic salmon. Front Microbiol. 2018;9:2877. doi:10.3389/fmicb.2018.02877 30542335 PMC6277808

[81] Pisu D , Huang L , Narang V , et al. Single cell analysis of *M. tuberculosis* phenotype and macrophage lineages in the infected lung. J Exp Med. 2021;218(9):e20210615. doi:10.1084/jem.20210615 34292313 PMC8302446

[82] Bost P , Giladi A , Liu Y , et al. Host‐viral infection maps reveal signatures of severe COVID‐19 patients. Cell. 2020;181(7):1475‐1488.e12. doi:10.1016/j.cell.2020.05.006 32479746 PMC7205692

[83] Gervais O , Gratacap R , Papadopoulou A , Houston RD , Hassan MA , Robledo D . Understanding host response to infectious salmon anaemia virus in an Atlantic salmon cell line using single‐cell RNA sequencing. bioRxiv. 10.1101/2022.01.04.474990 PMC10061729 36991327

[84] Mugimba KK , Byarugaba DK , Mutoloki S , Evensen Ø , Munang'andu HM . Challenges and solutions to viral diseases of finfish in marine aquaculture. Pathogens. 2021;10(6):673. doi:10.3390/pathogens10060673 34070735 PMC8227678

[85] Blix TB , Dalmo RA , Wargelius A , Myhr AI . Genome editing on finfish: current status and implications for sustainability. Rev Aquac. 2021;13(4):2344‐2363. doi:10.1111/raq.12571

[86] Gratacap RL , Wargelius A , Edvardsen RB , Houston RD . Potential of genome editing to improve aquaculture breeding and production. Trends Genet. 2019;35(9):672‐684. doi:10.1016/j.tig.2019.06.006 31331664

[87] Wargelius A . Application of genome editing in aquatic farm animals: Atlantic salmon. Transgenic Res. 2019;28(2):101‐105. doi:10.1007/s11248-019-00163-0 31321691

[88] Yang Z , Yu Y , Tay YX , Yue GH . Genome editing and its applications in genetic improvement in aquaculture. Rev Aquac. 2022;14(1):178‐191. doi:10.1111/raq.12591

[89] Gui T , Zhang J , Song F , et al. CRISPR/Cas9‐mediated genome editing and mutagenesis of EcChi4 in *Exopalaemon carinicauda* . G3 (Bethesda). 2016;6(11):3757‐3764. doi:10.1534/g3.116.034082 27605521 PMC5100874

[90] Yu H , Li H , Li Q , Xu R , Yue C , Du S . Targeted gene disruption in pacific oyster based on CRISPR/Cas9 ribonucleoprotein complexes. Mar Biotechnol. 2019;21(3):301‐309. doi:10.1007/s10126-019-09885-y 30810831

[91] Replogle JM , Saunders RA , Pogson AN , et al. Mapping information‐rich genotype‐phenotype landscapes with genome‐scale Perturb‐seq. Cell. 2022;185(14):2559‐2575.e28. doi:10.1016/j.cell.2022.05.013 35688146 PMC9380471

[92] Dixit A , Parnas O , Li B , et al. Perturb‐seq: dissecting molecular circuits with scalable single‐cell RNA profiling of pooled genetic screens. Cell. 2016;167(7):1853‐1866.e17. doi:10.1016/j.cell.2016.11.038 27984732 PMC5181115

[93] Datlinger P , Rendeiro AF , Schmidl C , et al. Pooled CRISPR screening with single‐cell transcriptome readout. Nat Methods. 2017;14(3):297‐301. doi:10.1038/nmeth.4177 28099430 PMC5334791

[94] Jaitin DA , Weiner A , Yofe I , et al. Dissecting immune circuits by linking CRISPR‐pooled screens with single‐cell RNA‐seq. Cell. 2016;167(7):1883‐1896.e15. doi:10.1016/j.cell.2016.11.039 27984734

[95] Alda‐Catalinas C , Bredikhin D , Hernando‐Herraez I , et al. A single‐cell transcriptomics CRISPR‐activation screen identifies epigenetic regulators of the zygotic genome activation program. Cell Syst. 2020;11(1):25‐41.e9. doi:10.1016/j.cels.2020.06.004 32634384 PMC7383230

[96] Mehravar M , Shirazi A , Nazari M , Banan M . Mosaicism in CRISPR/Cas9‐mediated genome editing. Dev Biol. 2019;445(2):156‐162. doi:10.1016/j.ydbio.2018.10.008 30359560

[97] Fatsini E , González W , Ibarra‐Zatarain Z , Napuchi J , Duncan NJ . The presence of wild Senegalese sole breeders improves courtship and reproductive success in cultured conspecifics. Aquaculture. 2020;519:734922. doi:10.1016/j.aquaculture.2020.734922

[98] Ofelio C , Guarniero I , Cariani A , et al. Monitoring of common sole *Solea solea* (L) captive broodstock from Northern Adriatic Sea over consecutive spawning seasons. Aquac Rep. 2020;18:100495. doi:10.1016/j.aqrep.2020.100495

[99] Budd AM , Banh QQ , Domingos JA , Jerry DR . Sex control in fish: approaches, challenges and opportunities for aquaculture. J Mar Sci Eng. 2015;3(2):329‐355. doi:10.3390/jmse3020329

[100] Breton S , Capt C , Guerra D , Stewart D . Sex‐determining mechanisms in bivalves. In: Leonard JL , ed. Transitions Between Sexual Systems: Understanding the Mechanisms of, and Pathways Between, Dioecy, Hermaphroditism and Other Sexual Systems. Springer International Publishing; 2018:165‐192. doi:10.1007/978-3-319-94139-4_6

[101] Toyota K , Miyakawa H , Hiruta C , et al. Sex determination and differentiation in Decapod and Cladoceran crustaceans: an overview of endocrine regulation. Genes. 2021;12(2):305. doi:10.3390/genes12020305 33669984 PMC7924870

[102] Stévant I , Nef S . Single cell transcriptome sequencing: a new approach for the study of mammalian sex determination. Mol Cell Endocrinol. 2018;468:11‐18. doi:10.1016/j.mce.2018.01.013 29371022

[103] Hollander‐Cohen L , Golan M , Levavi‐Sivan B . Differential regulation of gonadotropins as revealed by transcriptomes of distinct LH and FSH cells of fish pituitary. Int J Mol Sci. 2021;22(12):6478. doi:10.3390/ijms22126478 34204216 PMC8234412

[104] Siddique K , Ager‐Wick E , Fontaine R , Weltzien FA , Henkel CV . Characterization of hormone‐producing cell types in the teleost pituitary gland using single‐cell RNA‐seq. Sci Data. 2021;8(1):279. doi:10.1038/s41597-021-01058-8 34711832 PMC8553774

[105] Mei J , Gui JF . Genetic basis and biotechnological manipulation of sexual dimorphism and sex determination in fish. Sci China Life Sci. 2015;58(2):124‐136. doi:10.1007/s11427-014-4797-9 25563981

[106] Lu T , Mar JC . Investigating transcriptome‐wide sex dimorphism by multi‐level analysis of single‐cell RNA sequencing data in ten mouse cell types. Biol Sex Differ. 2020;11(1):61. doi:10.1186/s13293-020-00335-2 33153500 PMC7643324

[107] Xue L , Jia D , Xu L , et al. Bulk and single‐cell RNA‐seq reveal the sexually dimorphic expression pattern of dmrtb1 in zig‐zag eel (*Mastacembelus armatus*). Aquaculture. 2021;545:737194. doi:10.1016/j.aquaculture.2021.737194

[108] Stöck M , Kratochvíl L , Kuhl H , et al. A brief review of vertebrate sex evolution with a pledge for integrative research: towards 'sexomics'. Philos Trans R Soc Lond B Biol Sci. 1832;2021(376):20200426. doi:10.1098/rstb.2020.0426 PMC8293304 34247497

[109] Martínez P , Viñas AM , Sánchez L , Díaz N , Ribas L , Piferrer F . Genetic architecture of sex determination in fish: applications to sex ratio control in aquaculture. Front Genet. 2014;5:340. doi:10.3389/fgene.2014.00340 25324858 PMC4179683

[110] Guo J , Sosa E , Chitiashvili T , et al. Single‐cell analysis of the developing human testis reveals somatic niche cell specification and fetal germline stem cell establishment. Cell Stem Cell. 2021;28(4):764‐778.e4. doi:10.1016/j.stem.2020.12.004 33453151 PMC8026516

[111] Mayère C , Neirijnck Y , Sararols P , et al. Single‐cell transcriptomics reveal temporal dynamics of critical regulators of germ cell fate during mouse sex determination. FASEB J. 2021;35(4):e21452. doi:10.1096/fj.202002420R 33749946

[112] Estermann MA , Williams S , Hirst CE , et al. Insights into gonadal sex differentiation provided by single‐cell transcriptomics in the chicken embryo. Cell Rep. 2020;31(1):107491. doi:10.1016/j.celrep.2020.03.055 32268081

[113] Jung KM , Seo M , Kim YM , Kim JL , Han JY . Single‐cell RNA sequencing revealed the heterogeneity of gonadal primordial germ cells in zebra finch (*Taeniopygia guttata*). Front Cell Dev Biol. 2021;9:791335. doi:10.3389/fcell.2021.791335 34957119 PMC8695979

[114] Jin YH , Robledo D , Hickey JM , McGrew MJ , Houston RD . Surrogate broodstock to enhance biotechnology research and applications in aquaculture. Biotechnol Adv. 2021;49:107756. doi:10.1016/j.biotechadv.2021.107756 33895331 PMC8192414

[115] Fraslin C , Yáñez JM , Robledo D , Houston RD . The impact of genetic relationship between training and validation populations on genomic prediction accuracy in Atlantic salmon. Aquac Rep. 2022;23:101033. doi:10.1016/j.aqrep.2022.101033

[116] Calderon D , Bhaskar A , Knowles DA , et al. Inferring relevant cell types for complex traits by using single‐cell gene expression. Am J Hum Genet. 2017;101(5):686‐699. doi:10.1016/j.ajhg.2017.09.009 29106824 PMC5673624

[117] Watanabe K , Umićević Mirkov M , de Leeuw CA , van den Heuvel MP , Posthuma D . Genetic mapping of cell type specificity for complex traits. Nat Commun. 2019;10(1):3222. doi:10.1038/s41467-019-11181-1 31324783 PMC6642112

[118] Maria M , Pouyanfar N , Örd T , Kaikkonen MU . The power of single‐cell RNA sequencing in eQTL discovery. Genes. 2022;13(3):502. doi:10.3390/genes13030502 35328055 PMC8949403

[119] Maurano MT , Humbert R , Rynes E , et al. Systematic localization of common disease‐associated variation in regulatory DNA. Science. 2012;337(6099):1190‐1195. doi:10.1126/science.1222794 22955828 PMC3771521

[120] Yazar S , Alquicira‐Hernandez J , Wing K , et al. Single‐cell eQTL mapping identifies cell type‐specific genetic control of autoimmune disease. Science. 2022;376(6589):eabf3041. doi:10.1126/science.abf3041 35389779

[121] Nathan A , Asgari S , Ishigaki K , et al. Single‐cell eQTL models reveal dynamic T cell state dependence of disease loci. Nature. 2022;606(7912):120‐128. doi:10.1038/s41586-022-04713-1 35545678 PMC9842455

[122] Dong S , Boyle AP . Prioritization of regulatory variants with tissue‐specific function in the non‐coding regions of human genome. Nucleic Acids Res. 2021;50(1):e6. doi:10.1093/nar/gkab924 PMC8754628 34648033

[123] Bakken TE , Hodge RD , Miller JA , et al. Single‐nucleus and single‐cell transcriptomes compared in matched cortical cell types. PLoS One. 2018;13(12):e0209648. doi:10.1371/journal.pone.0209648 30586455 PMC6306246

[124] Thrupp N , Sala Frigerio C , Wolfs L , et al. Single‐nucleus RNA‐Seq is not suitable for detection of microglial activation genes in humans. Cell Rep. 2020;32(13):108189. doi:10.1016/j.celrep.2020.108189 32997994 PMC7527779

[125] Ding J , Adiconis X , Simmons SK , et al. Systematic comparison of single‐cell and single‐nucleus RNA‐sequencing methods. Nat Biotechnol. 2020;38(6):737‐746. doi:10.1038/s41587-020-0465-8 32341560 PMC7289686

[126] Wu L , Ferger KE , Lambert JD . Gene expression does not support the developmental hourglass model in three animals with spiralian development. Mol Biol Evol. 2019;36(7):1373‐1383. doi:10.1093/molbev/msz065 30895314

[127] Andrews TS , Atif J , Liu JC , et al. Single‐cell, single‐nucleus, and spatial RNA sequencing of the human liver identifies cholangiocyte and mesenchymal heterogeneity. Hepatol Commun. 2022;6(4):821‐840. doi:10.1002/hep4.1854 34792289 PMC8948611

[128] Selewa A , Dohn R , Eckart H , et al. Systematic comparison of high‐throughput single‐cell and single‐nucleus transcriptomes during cardiomyocyte differentiation. Sci Rep. 2020;10(1):1535. doi:10.1038/s41598-020-58327-6 32001747 PMC6992778

[129] Gupta A , Shamsi F , Altemose N , et al. Characterization of transcript enrichment and detection bias in single‐nucleus RNA‐seq for mapping of distinct human adipocyte lineages. Genome Res. 2022;32(2):242‐257. doi:10.1101/gr.275509.121 35042723 PMC8805720

[130] Nguyen QH , Pervolarakis N , Nee K , Kessenbrock K . Experimental considerations for single‐cell RNA sequencing approaches. Front Cell Dev Biol. 2018;6:108. doi:10.3389/fcell.2018.00108 30234113 PMC6131190

[131] Gao R , Kim C , Sei E , et al. Nanogrid single‐nucleus RNA sequencing reveals phenotypic diversity in breast cancer. Nat Commun. 2017;8(1):228. doi:10.1038/s41467-017-00244-w 28794488 PMC5550415

[132] Habib N , Avraham‐Davidi I , Basu A , et al. Massively parallel single‐nucleus RNA‐seq with DroNc‐seq. Nat Methods. 2017;14(10):955‐958. doi:10.1038/nmeth.4407 28846088 PMC5623139

[133] Wu H , Kirita Y , Donnelly EL , Humphreys BD . Advantages of single‐nucleus over single‐cell RNA sequencing of adult kidney: rare cell types and novel cell states revealed in fibrosis. J Am Soc Nephrol. 2019;30(1):23‐32. doi:10.1681/ASN.2018090912 30510133 PMC6317600

[134] Ramachandran P , Dobie R , Wilson‐Kanamori JR , et al. Resolving the fibrotic niche of human liver cirrhosis at single‐cell level. Nature. 2019;575(7783):512‐518. doi:10.1038/s41586-019-1631-3 31597160 PMC6876711

[135] Grindberg RV , Yee‐Greenbaum JL , McConnell MJ , et al. RNA‐sequencing from single nuclei. Proc Natl Acad Sci U S A. 2013;110(49):19802‐19807. doi:10.1073/pnas.1319700110 24248345 PMC3856806

[136] Sen A , Kallos MS , Behie LA . New tissue dissociation protocol for scaled‐up production of neural stem cells in suspension bioreactors. Tissue Eng. 2004;10(5‐6):904‐913. doi:10.1089/1076327041348554 15265308

[137] Volovitz I , Shapira N , Ezer H , et al. A non‐aggressive, highly efficient, enzymatic method for dissociation of human brain‐tumors and brain‐tissues to viable single‐cells. BMC Neurosci. 2016;17(1):30. doi:10.1186/s12868-016-0262-y 27251756 PMC4888249

[138] Bresciani E , Broadbridge E , Liu PP . An efficient dissociation protocol for generation of single cell suspension from zebrafish embryos and larvae. MethodsX. 2018;5:1287‐1290. doi:10.1016/j.mex.2018.10.009 30364607 PMC6197777

[139] Tsuji K , Ojima M , Otabe K , et al. Effects of different cell‐detaching methods on the viability and cell surface antigen expression of synovial mesenchymal stem cells. Cell Transplant. 2017;26(6):1089‐1102. doi:10.3727/096368917X694831 28139195 PMC5657749

[140] Lacar B , Linker SB , Jaeger BN , et al. Nuclear RNA‐seq of single neurons reveals molecular signatures of activation. Nat Commun. 2016;7:11022. doi:10.1038/ncomms11022 27090946 PMC4838832

[141] Van Den Brink SC , Sage F , Vértesy Á , et al. Single‐cell sequencing reveals dissociation‐induced gene expression in tissue subpopulations. Nat Methods. 2017;14(10):935‐936. doi:10.1038/nmeth.4437 28960196

[142] Machado L , Relaix F , Mourikis P . Stress relief: emerging methods to mitigate dissociation‐induced artefacts. Trends Cell Biol. 2021;31(11):888‐897. doi:10.1016/j.tcb.2021.05.004 34074577

[143] Usoskin D , Furlan A , Islam S , et al. Unbiased classification of sensory neuron types by large‐scale single‐cell RNA sequencing. Nat Neurosci. 2015;18(1):145‐153. doi:10.1038/nn.3881 25420068

[144] Krishnaswami SR , Grindberg RV , Novotny M , et al. Using single nuclei for RNA‐seq to capture the transcriptome of postmortem neurons. Nat Protoc. 2016;11(3):499‐524. doi:10.1038/nprot.2016.015 26890679 PMC4941947

[145] Adam M , Potter AS , Potter SS . Psychrophilic proteases dramatically reduce single‐cell RNA‐seq artifacts: a molecular atlas of kidney development. Development. 2017;144(19):3625‐3632. doi:10.1242/dev.151142 28851704 PMC5665481

[146] O'Flanagan CH , Campbell KR , Zhang AW , et al. Dissociation of solid tumor tissues with cold active protease for single‐cell RNA‐seq minimizes conserved collagenase‐associated stress responses. Genome Biol. 2019;20(1):210. doi:10.1186/s13059-019-1830-0 31623682 PMC6796327

[147] Alles J , Karaiskos N , Praktiknjo SD , et al. Cell fixation and preservation for droplet‐based single‐cell transcriptomics. BMC Biol. 2017;15(1):44. doi:10.1186/s12915-017-0383-5 28526029 PMC5438562

[148] Guillaumet‐Adkins A , Rodríguez‐Esteban G , Mereu E , et al. Single‐cell transcriptome conservation in cryopreserved cells and tissues. Genome Biol. 2017;18(1):45. doi:10.1186/s13059-017-1171-9 28249587 PMC5333448

[149] García‐Castro H , Kenny NJ , Iglesias M , et al. ACME dissociation: a versatile cell fixation‐dissociation method for single‐cell transcriptomics. Genome Biol. 2021;22(1):89. doi:10.1186/s13059-021-02302-5 33827654 PMC8028764

[150] Ayhan F , Douglas C , Lega BC , Konopka G . Nuclei isolation from surgically resected human hippocampus. STAR Protoc. 2021;2(4):100844. doi:10.1016/j.xpro.2021.100844 34585170 PMC8455478

[151] Corces MR , Trevino AE , Hamilton EG , et al. An improved ATAC‐seq protocol reduces background and enables interrogation of frozen tissues. Nat Methods. 2017;14(10):959‐962. doi:10.1038/nmeth.4396 28846090 PMC5623106

[152] Drokhlyansky E , Smillie CS , Van Wittenberghe N , et al. The human and mouse enteric nervous system at single‐cell resolution. Cell. 2020;182(6):1606‐1622.e23. doi:10.1016/j.cell.2020.08.003 32888429 PMC8358727

[153] Melms JC , Biermann J , Huang H , et al. A molecular single‐cell lung atlas of lethal COVID‐19. Nature. 2021;595(7865):114‐119. doi:10.1038/s41586-021-03569-1 33915568 PMC8814825

[154] Andrews TS , Yu Kiselev V , Hemberg M . Statistical methods for single‐cell RNA‐sequencing. Handbook of Statistical Genomics. John Wiley & Sons, Ltd; 2019:735‐720. doi:10.1002/9781119487845.ch26

[155] Alfieri JM , Wang G , Jonika MM , Gill CA , Blackmon H , Athrey GN . A primer for single‐cell sequencing in non‐model organisms. Genes. 2022;13(2):380. doi:10.3390/genes13020380 35205423 PMC8872538

[156] Nip KM , Chiu R , Yang C , et al. RNA‐bloom enables reference‐free and reference‐guided sequence assembly for single‐cell transcriptomes. Genome Res. 2020;30(8):1191‐1200. doi:10.1101/gr.260174.119 32817073 PMC7462077

[157] Botvinnik OB , Vemuri VNP , Pierce NT , et al. Single‐cell transcriptomics for the 99.9% of species without reference genomes. bioRxiv. doi:10.1101/2021.07.09.450799

[158] Redmond AK , Zou J , Secombes CJ , Macqueen DJ , Dooley H . Discovery of all three types in cartilaginous fishes enables phylogenetic resolution of the origins and evolution of interferons. Front Immunol. 2019;10:1558. doi:10.3389/fimmu.2019.01558 31354716 PMC6640115

[159] Macqueen DJ , Johnston IA . A well‐constrained estimate for the timing of the salmonid whole genome duplication reveals major decoupling from species diversification. Proc Biol Sci. 2014;281(1778):20132881. doi:10.1098/rspb.2013.2881 24452024 PMC3906940

[160] Lien S , Koop BF , Sandve SR , et al. The Atlantic salmon genome provides insights into rediploidization. Nature. 2016;533(7602):200‐205. doi:10.1038/nature17164 27088604 PMC8127823

[161] Xu P , Xu J , Liu G , et al. The allotetraploid origin and asymmetrical genome evolution of the common carp *Cyprinus carpio* . Nat Commun. 2019;10(1):4625. doi:10.1038/s41467-019-12644-1 31604932 PMC6789147

[162] Luo J , Chai J , Wen Y , et al. From asymmetrical to balanced genomic diversification during rediploidization: Subgenomic evolution in allotetraploid fish. Sci Adv. 2020;6(22):eaaz7677. doi:10.1126/sciadv.aaz7677 32766441 PMC7385415

[163] Gabaldón T , Koonin EV . Functional and evolutionary implications of gene orthology. Nat Rev Genet. 2013;14(5):360‐366. doi:10.1038/nrg3456 23552219 PMC5877793

[164] Durinck S , Spellman PT , Birney E , Huber W . Mapping identifiers for the integration of genomic datasets with the R/Bioconductor package biomaRt. Nat Protoc. 2009;4(8):1184‐1191. doi:10.1038/nprot.2009.97 19617889 PMC3159387

[165] Emms DM , Kelly S . OrthoFinder: phylogenetic orthology inference for comparative genomics. Genome Biol. 2019;20(1):238. doi:10.1186/s13059-019-1832-y 31727128 PMC6857279

[166] Wang J , Sun H , Jiang M , et al. Tracing cell‐type evolution by cross‐species comparison of cell atlases. Cell Rep. 2021;34(9):108803. doi:10.1016/j.celrep.2021.108803 33657376

[167] Kaminow B , Yunusov D , Dobin A . STARsolo: accurate, fast and versatile mapping/quantification of single‐cell and single‐nucleus RNA‐seq data. bioRxiv. doi:10.1101/2021.05.05.442755

[168] Srivastava A , Malik L , Sarkar H , Patro R . A Bayesian framework for inter‐cellular information sharing improves dscRNA‐seq quantification. Bioinformatics. 2020;36(1):i292‐i299. doi:10.1093/bioinformatics/btaa450 32657394 PMC7355277

[169] Brüning RS , Tombor L , Schulz MH , Dimmeler S , John D . Comparative analysis of common alignment tools for single‐cell RNA sequencing. GigaScience. 2022;11:giac001. doi:10.1093/gigascience/giac001 35084033 PMC8848315

[170] Lun ATL , Riesenfeld S , Andrews T , et al. EmptyDrops: distinguishing cells from empty droplets in droplet‐based single‐cell RNA sequencing data. Genome Biol. 2019;20(1):63. doi:10.1186/s13059-019-1662-y 30902100 PMC6431044

[171] Hay SB , Ferchen K , Chetal K , Grimes HL , Salomonis N . The human cell atlas bone marrow single‐cell interactive web portal. Exp Hematol. 2018;68:51‐61. doi:10.1016/j.exphem.2018.09.004 30243574 PMC6296228

[172] Hao Y , Hao S , Andersen‐Nissen E , et al. Integrated analysis of multimodal single‐cell data. Cell. 2021;184(13):3573‐3587.e29. doi:10.1016/j.cell.2021.04.048 34062119 PMC8238499

[173] Wolf FA , Angerer P , Theis FJ . SCANPY: large‐scale single‐cell gene expression data analysis. Genome Biol. 2018;19(1):15. doi:10.1186/s13059-017-1382-0 29409532 PMC5802054

[174] Young MD , Behjati S . SoupX removes ambient RNA contamination from droplet‐based single‐cell RNA sequencing data. GigaScience. 2020;9(12):1‐10. doi:10.1093/gigascience/giaa151 PMC7763177 33367645

[175] Fleming SJ , Chaffin MD , Arduini A , et al. Unsupervised removal of systematic background noise from droplet‐based single‐cell experiments using CellBender. bioRxiv. 10.1101/791699 37550580

[176] Yang S , Corbett SE , Koga Y , et al. Decontamination of ambient RNA in single‐cell RNA‐seq with DecontX. Genome Biol. 2020;21(1):57. doi:10.1186/s13059-020-1950-6 32138770 PMC7059395

[177] Xi NM , Li JJ . Benchmarking computational doublet‐detection methods for single‐cell RNA sequencing data. Cell Syst. 2021;12(2):176‐194.e6. doi:10.1016/j.cels.2020.11.008 33338399 PMC7897250

[178] Germain PL , Lun A , Garcia Meixide C , Macnair W , Robinson MD . Doublet identification in single‐cell sequencing data using scDblFinder. F1000Res. 2021;10:979. doi:10.12688/f1000research.73600.2 35814628 PMC9204188

[179] Hou W , Ji Z , Ji H , Hicks SC . A systematic evaluation of single‐cell RNA‐sequencing imputation methods. Genome Biol. 2020;21(1):218. doi:10.1186/s13059-020-02132-x 32854757 PMC7450705

[180] Andrews TS , Hemberg M . False signals induced by single‐cell imputation. F1000Res. 2018;7:1740. doi:10.12688/f1000research.16613.2 30906525 PMC6415334

[181] Monteiro JP , Rodor J , Caudrillier A , et al. MIR503HG loss promotes endothelial‐to‐mesenchymal transition in vascular disease. Circ Res. 2021;128(8):1173‐1190. doi:10.1161/CIRCRESAHA.120.318124 33703914 PMC7610629

[182] Petegrosso R , Li Z , Kuang R . Machine learning and statistical methods for clustering single‐cell RNA‐sequencing data. Brief Bioinform. 2020;21(4):1209‐1223. doi:10.1093/bib/bbz063 31243426

[183] Qi R , Ma A , Ma Q , Zou Q . Clustering and classification methods for single‐cell RNA‐sequencing data. Brief Bioinform. 2020;21(4):1196‐1208. doi:10.1093/bib/bbz062 31271412 PMC7444317

[184] Patterson‐Cross RB , Levine AJ , Menon V . Selecting single cell clustering parameter values using subsampling‐based robustness metrics. BMC Bioinform. 2021;22(1):39. doi:10.1186/s12859-021-03957-4 PMC7852188 33522897

[185] Zheng HB , Doran BA , Kimler K , et al. A treatment‐naïve cellular atlas of pediatric Crohn's disease predicts disease severity and therapeutic response. medRxiv. doi:10.1101/2021.09.17.21263540

[186] Cakir B , Prete M , Huang N , van Dongen S , Pir P , Kiselev VY . Comparison of visualization tools for single‐cell RNAseq data. NAR Genom Bioinform. 2020;2(3):lqaa052. doi:10.1093/nargab/lqaa052 32766548 PMC7391988

[187] Rao A , Barkley D , França GS , Yanai I . Exploring tissue architecture using spatial transcriptomics. Nature. 2021;596(7871):211‐220. doi:10.1038/s41586-021-03634-9 34381231 PMC8475179

[188] Li Y , Zhou F , Yang Q , et al. Single‐cell sequencing reveals types of hepatopancreatic cells and haemocytes in black tiger shrimp (*Penaeus monodon*) and their molecular responses to ammonia stress. Front Immunol. 2022;13:883043. doi:10.3389/fimmu.2022.883043 35603188 PMC9114817

[189] Meng J , Zhang G , Wang WX . Functional heterogeneity of immune defenses in molluscan oysters *Crassostrea hongkongensis* revealed by high‐throughput single‐cell transcriptome. Fish Shellfish Immunol. 2022;120:202‐213. doi:10.1016/j.fsi.2021.11.027 34843943

[190] McCarthy DJ , Campbell KR , Lun ATL , Wills QF . Scater: pre‐processing, quality control, normalization and visualization of single‐cell RNA‐seq data in R. Bioinformatics. 2017;33(8):1179‐1186. doi:10.1093/bioinformatics/btw777 28088763 PMC5408845

[191] La Manno G , Soldatov R , Zeisel A , et al. RNA velocity of single cells. Nature. 2018;560(7719):494‐498. doi:10.1038/s41586-018-0414-6 30089906 PMC6130801

[192] Street K , Risso D , Fletcher RB , et al. Slingshot: cell lineage and pseudotime inference for single‐cell transcriptomics. BMC Genom. 2018;19(1):477. doi:10.1186/s12864-018-4772-0 PMC6007078 29914354

[193] Frishberg A , Peshes‐Yaloz N , Cohn O , et al. Cell composition analysis of bulk genomics using single‐cell data. Nat Methods. 2019;16(4):327‐332. doi:10.1038/s41592-019-0355-5 30886410 PMC6443043

[194] Jew B , Alvarez M , Rahmani E , et al. Accurate estimation of cell composition in bulk expression through robust integration of single‐cell information. Nat Commun. 2020;11:1971. doi:10.1038/s41467-020-15816-6 32332754 PMC7181686

[195] Efremova M , Vento‐Tormo M , Teichmann SA , Vento‐Tormo R . CellPhoneDB: inferring cell–cell communication from combined expression of multi‐subunit ligand–receptor complexes. Nat Protoc. 2020;15(4):1484‐1506. doi:10.1038/s41596-020-0292-x 32103204

[196] Lim CY , Wang H , Woodhouse S , et al. BTR: training asynchronous Boolean models using single‐cell expression data. BMC Bioinform. 2016;17(1):355. 10.1186/s12859-016-1235-y PMC5012073 27600248

